# Viral skin diseases in odontocete cetaceans: gross, histopathological, and molecular characterization of selected pathogens

**DOI:** 10.3389/fvets.2023.1188105

**Published:** 2023-09-08

**Authors:** Simone Segura-Göthlin, Antonio Fernández, Manuel Arbelo, Marisa Ana Andrada Borzollino, Idaira Felipe-Jiménez, Ana Colom-Rivero, Carla Fiorito, Eva Sierra

**Affiliations:** ^1^Veterinary Histology and Pathology, Atlantic Center for Cetacean Research, University Institute of Animal Health and Food Safety (IUSA), Veterinary School, University of Las Palmas de Gran Canaria (ULPGC), Las Palmas, Spain; ^2^Centro para el Estudio de Sistemas Marinos, Consejo Nacional de Investigaciones Científicas y Técnicas (CESIMAR-CONICET), Puerto Madryn, Chubut, Argentina

**Keywords:** cetacean poxvirus, coinfection, herpesvirus, histopathology, molecular diagnosis, morbillivirus, skin lesions

## Abstract

Fifty-five skin lesions from 31 stranded cetaceans along the Canary coasts (2011–2021) were submitted to macroscopic, histological, and molecular analyses to confirm infection by cetacean poxvirus, herpesvirus and cetacean morbillivirus. They were macroscopically categorized into eight categories with respective subcategories according to their color, shape, size, and consistency. Cetacean poxvirus was detected in 54.54% of the skin lesions through real-time and conventional PCRs based on the DNA polymerase gene. Additionally, herpesvirus and morbillivirus were currently detected from 43.63 and 1.82% of the cutaneous lesions, respectively. Coinfection of poxvirus and herpesvirus was detected in nine of them (16.36%), which makes the present study the first to report coinfection by both pathogens in skin lesions in cetaceans. A plausible approach to histopathological characterization of poxvirus-and herpesvirus-positive skin lesions was established. Hyperkeratosis, acanthosis, ballooning degeneration, and intracytoplasmic inclusion bodies in vacuolized keratinocytes through the stratum spinosum were common findings in poxvirus skin lesions. Alphaherpesvirus was associated with a prominent acanthotic epidermis, moderate necrosis, multifocal dyskeratosis, and irregular keratinocytes with both cellular and nuclei pleomorphism. The common histopathological findings of both pathogens were observed in coinfection lesions. However, those associated with herpesvirus were considerably more remarkable. Relationships between molecular and microscopic findings were observed for the lesions that showed tattoo-like and tortuous patterns. Further multidisciplinary diagnostic studies of infected skin lesions are needed to understand the epidemiology of these emerging infectious diseases.

## 1. Introduction

Cetaceans have long life spans, resident and transient strategies, and high trophic levels that make them promising as sentinels that reflect large-scale aquatic ecosystem health (1–4). The long-term investigations of these marine mammals in the past two decades have facilitated the documentation in wild populations of several diseases, including those caused by emerging or re-emerging pathogens (5, 6). Researchers consider cetacean epidermal conditions as useful for evaluating species health and environmental status (2, 7, 8). Skin diseases are among the most well-documented diseases that affect cetacean species globally (9). Apart from their high visibility, they are of particular scientific interest for several reasons: (1) some microorganisms affecting the skin are considered opportunistic, as they invade and infect pre-existing wounds, leading to the progression of distinctive skin lesions or systemic infections (10–12); (2) the prevalence and persistence of skin diseases in these marine mammals relates to host immunologic dysfunction resulting from chronic exposure to anthropogenic factors, distress, and other infectious diseases (13–15); and (3) they usually involve a broad spectrum of pathogens (16).

Several cutaneous lesions have been associated with viruses in free-ranging cetaceans, including the cetacean poxvirus (CePV) (17). CePV causes the most widely reported and globally prevalent skin disease and is typically diagnosed through visual assessment (18–20). CePV has a distinctive clinical presentation characterized by flat or slightly raised hyperpigmented oval patches that may be solitary or coalescing and give the appearance of “ring-like” lesions (21). However, CePV can also present with an irregular stippled pattern, commonly referred to as a “tattoo” lesion, which prompted the categorization of this disease as tattoo skin disease (TSD) (22). CePV may reflect generalized immune suppression in cetacean populations, making it a potential indicator of cetacean health (4, 23, 24). Herpesvirus (HV) infections in cetaceans are more commonly associated with systemic infections (25–27) and encephalitis (28–30) related to the *Alphaherpesvirinae* subfamily (alphaherpesvirus) (31). Nevertheless, gammaherpesviruses have also been detected in genital and skin lesions with different manifestations in cetaceans (32), ranging from flat oval lesions and proliferative wounds to raised verrucous nodules and plaque-like lesions, respectively (11). Regarding skin disorders, HV in cetaceans has been associated with different types of dermatitis, such as proliferative, fibrinosuppurative, and necrotizing dermatitis (33–35). Viral skin diseases have been less frequently associated with papillomaviruses that cause proliferative nodules (18), calicivirus-inducing vesicular disease (36–38), and morbilliviruses skin lesions along with severe respiratory, nervous, and immune impairments (6, 39, 40). Morbilliviruses are among the most significant emerging pathogens of cetaceans globally and cause lethal disease outbreaks with extensive geographic distributions among very large host populations of cetaceans (39, 41).

Nevertheless, skin lesion assessments are challenging for free-ranging cetaceans because of their limited accessibility in the wild and the costly and time-consuming investments required (42–44). Hence, most studies have relied on long-term photographic surveys to evaluate the progression and course of skin diseases (13, 45, 46). Observational or photographic surveys are, however, considered suboptimal, and ancillary diagnostic tests are required to determine the causative agent of a skin disorder even when the macroscopic manifestation is assumed to be characteristic or pathognomonic of a specific etiology (47–49). Accordingly, most studies strictly associate CePV with typical tattoo-like lesions, disregarding other possible skin manifestations that can be triggered by this virus. This leads to limited genomic information with which to correctly designate this pathogen (20, 50, 51). Likewise, restricting the detection of this pathogen from tattoo-like lesions reduces the probability of identifying co-infections from macroscopically different lesions. On this premise, the detection of pathogens from skin lesions would enable genomic characterization and phylogenetic analysis and facilitate a better understanding of the epidemiology of these pathogens.

The aim of the present study is a complete molecular screening of poxvirus and other viruses, such as herpesvirus and cetacean morbillivirus, in various skin disorders from stranded cetaceans in the Canary Islands. Additionally, macroscopic, histological, and molecular examinations, in conjunction with phylogenetic analysis, were performed to provide insights about these emerging infectious skin diseases.

## 2. Materials and methods

This was a retrospective study, and skin samples were selected from cetaceans with good to moderate states of preservation, and/or the collection of both formalin-fixed and fresh unfixed portions from each skin sample. Accordingly, skin samples (*n* = 55) from 31 cetaceans stranded on the coast of the Canary Archipelago, Spain, from March 2011 to May 2021 were analyzed. Six different species of cetaceans were included in the present study, including striped dolphins (*Stenella coeruleoalba*; *N* = 10), Atlantic spotted dolphins (*Stenella frontalis*; *N* = 9), common dolphins (*Delphinus delphis*; *N* = 4), common bottlenose dolphins (*Tursiops truncatus*; *N* = 3), short-finned pilot whales (*Globicephala macrorhynchus*; *N* = 3), and Risso’s dolphins (*Grampus griseus*; *N* = 2). All study samples were subjected to standardized necropsies, and the decomposition code, conservation methods, and other data (including sex and age) for each animal were obtained according to standard guidelines (52–55). Five decomposition codes were established: code 1 (extremely fresh) to code 5 (mummified or skeletal) (55). Most animals had a good state of preservation (code 2), while four animals were euthanized (56, 57) because of a poor prognosis and provided extremely fresh carcasses (code 1). Nevertheless, for management reasons, it was not always possible to perform necropsies of individuals preserved at room temperature, and some animals were kept frozen to avoid further decomposition prior to necropsy. Based on the total body length and histological gonadal development, the age categories were classified as follows: neonate, calf, juvenile, and adult (58, 59). Additionally, stranding and epidemiological information (type, location, and date) were also systematically recorded and have been summarized in Supplementary Table S1. Notably, four animals in the present study have been previously published; poxvirus was detected in three of these animals (cases 2, 27, and 30; CETS 601, 1,151, and 1,173, respectively) and herpesvirus was detected in another (case 25; CET 1103). During necropsies, formalin-fixed and fresh unfixed samples of representative tissues, including skin samples, were collected for histopathologic and molecular analysis, respectively (60). Fixed tissues were submitted in 10% neutral buffered formalin solution, processed, embedded in paraffin blocks, and sectioned into 5 μm slices before staining with hematoxylin and eosin (HE). Fresh unfixed samples were stored at −80°C before being selectively submitted for virological testing and mycological and bacteriological analyses. For the latter, slices were cultured on Sabouraud agar and morphologic colony identification was performed along with routine culture and surface plating on Columbia blood agar; the API system was used for preliminary identification of isolates (54, 60). The epibionts, ectoparasites, and endoparasites were preserved in 70% alcohol for parasitological analysis. The identification relied on macroscopic, submacroscopic, and histologic features (60, 61).

### 2.1. Macroscopic analysis of skin lesions

All skin lesions were described, measured, and photographed. Their locations on the body were recorded along with their macroscopic appearance, color, shape, and consistency.

### 2.2. Molecular analysis of skin lesions

For each study animal, 0.5 g of fresh-frozen skin sample was added to 500 μl 1X cell lysis buffer (Cell Signaling Technology, United States) and 4.5 ml of diethylpyrocarbonate (DEPC)-treated water (Ambion, Invitrogen) for two consecutive rounds of mechanical homogenization at 3549 ×*g* with a 30-s rest interval in a Precellys 24 tissue homogenizer (Bertin Technologies SAS, France). The homogenized samples were centrifuged at 2163 ×*g* for 15 min at 4°C in a high-speed refrigerated benchtop centrifuge (Megafuge series, Thermo Fisher Scientific, Waltham, MA, United States). Total DNA/RNA extraction from each 300 μl macerated sample was performed using a QuickGene Mini 80 nucleic acid isolation machine (QuickGene, Kurabo, Japan) according to the manufacturer’s instructions, with a slight modification: RNA carrier (Applied Biosystems, Thermo Fisher Scientific) was added during the lysis step as previously described (62).

CePV-1 molecular detection from 55 extracted samples was performed using two different assays. First, semi-quantitative polymerase chain reaction (sqPCR) based on SYBR green was used to amplify a conserved region (150 bp) of the odontocete poxvirus DNA polymerase gene using the degenerate primer sets designed by Sacristán and coworkers (20). To assess specificity, a conventional PCR amplification of the 543-bp fragment from the *Chordopoxvirinae* subfamily (capri-, sui-, cervid-, and ortho-poxvirus) DNA polymerase gene of the qPCR CePV positive samples was also performed using the primer sequences originally designed by Bracht and collaborators (50). PCR products (5 μl per sample) were read on a 2% agarose electrophoresis gel containing GelRed (Biotium, Inc., California, United States).

Panherpesvirus conventional nested PCR was performed for HV detection using the universal HV nested PCR protocol originally developed by VanDevanter and coworkers (63). Additionally, to obtain semi-quantitative data on viral loads of each sample, a nested SYBR Green sqPCR for HV detection was carried out using the same degenerate primers as above to amplify a 200-bp region of the DNA polymerase gene as in conventional PCR (29). A 4-μL aliquot from the DNA extraction was amplified in a mixture containing 10 μl of 2X SsoAdvanced Universal SYBR Green Supermix with a high-fidelity Taq DNA polymerase based on Bio-Rad’s patented Sso7d fusion protein technology (Bio-Rad Laboratories, Inc., California, CA, United States), 250 nM of each primer, 1x GC-RICH solution (Roche Diagnostics S.L., Barcelona, Spain), and nuclease-free water to bring the final volume to 20 μL. The reactions were set for 3 min of polymerase activation at 98°C, followed by 45 amplification cycles, each comprising a denaturation step at 95°C for 15 s, an annealing step at 46°C for 30 s, and an elongation step at 72°C for 1 min. The final cycle was composed of an extended elongation at 72°C for 7 min. Thereafter, 5 μL of the amplicons from the second PCR were read by 2% agarose gel electrophoresis to corroborate the sq-PCR results.

Furthermore, total RNA extracted from the 55 skin samples was submitted for molecular detection of the Cetacean Morbillivirus (CeMV) through sq-PCR using primers targeting highly conserved fragments of the phosphoprotein gene (205 bp), as previously described (41). Two negative and positive controls (for extraction and amplification) were included in each protocol.

PCR products were purified using a Real Clean spin kit (REAL, Durviz, S.L., Valencia, Spain) for sequencing (Secugen S.L., Madrid, Spain). Sequencing used 1 μl (5 μM) of each of the following primers: Odontopox-F and Odontopox-R for CePV-1 (20), TGV (internal forward) and IYG (internal reverse) for HV (63), and PAN-F and PAN-R for CeMV (41). Amplicon identities were confirmed with BLAST.^1^

The cycle threshold (Ct) values for the CePV and HV sq-PCRs, which consisted of the target-specific amplification signals, were determined to assess viral loads and the risk of transmission and recovery (64). Late Cts (typically cycles 30–45) are near the limit of detection and are considered marginally positive (65). Ct values are inversely related to viral loads; greater concentrations of viral genetic material require fewer cycles of amplification (66). Nevertheless, caution should be taken when evaluating this factor as poor DNA extraction and/or nucleic acid degradation can affect results. Melting curves were used to confirm the amplification of the dsDNA products.

### 2.3. Phylogenetic analysis

The sequences of HV and CePV were aligned (excluding primers) with the Clustal W algorithm using MEGA X software (Pennsylvania, PA, United States) (67, 68). A total of 99 and 29 HV and CePV-1 nucleotide sequences, respectively, were recovered from GenBank to construct the phylogenetic trees. Both trees were established from deduced nucleotide sequences using the Maximum Likelihood Method. Accordingly, for HV, the Tamura 2-parameter model with a discrete Gamma distribution was used to model the evolutionary rate differences among sites (5 categories (+G, parameter = 0.7779)). The Tamura 3-parameter model with a Gamma parameter of 0.2836 was used for modeling the CePV tree (67). Bootstrap consensus trees were inferred from 500 replicates. Although branches corresponding to partitions reproduced in <50% of bootstrap replicates are collapsed, only values >70% were considered meaningful.

### 2.4. Histopathological analysis of skin lesions

Thirty-three of 55 (69.1%) skin lesions were considered for histologic analysis (including lesions that were positive and negative by a molecular test for any of the three pathogens). To accurately relate histopathological changes with the viruses involved, skin lesions that histologically showed coinfection by other etiological agents such as bacteria or protozoa (*n* = 6) were not considered. This also applied to skin lesions associated with traumatic wounds (*n* = 1). Carcasses that were too compromised to submit to freezing preservation (*n* = 7) or that were too advanced in decomposition code (*n* = 4) were not considered because of artifacts unavoidably induced by the freeze–thaw process and tissue autolysis. Moreover, samples from four skin lesions were not available for histopathological analysis.

The frequent histopathological findings associated with viral skin infections were graded as follows: absent (−), minimal (+), mild (++), moderate (+++), and severe (++++) (69). Plausible associations of histological observations with macroscopic appraisals, as well as molecular findings, were investigated.

Immunohistochemistry techniques (IHC) targeting HV and CeMV were also performed on respective positive skin lesions as complementary diagnostic assays. Thus, serial sections (3 μm thickness) were sliced and stained as previously described (29, 70). Appropriate positive and negative immunohistochemical controls were included for both IHCs.

## 3. Results

### 3.1. Macroscopic findings of skin lesions

The skin lesions were categorized as shown in Table 1. The most observed pattern was the tattoo-like oval shape lesion (TL-O), followed by black-fringed (BF) and white-fringed (WF) lesions. The remaining categories were rather equally reported, except the pale pattern (P), which was rarest. The lesions were predominantly on the heads and both flanks of cetaceans, though lesions were also found on the fins and the ventral regions. Generally, lesions were of different sizes, and animals rarely had multiple lesions. Twenty skin lesions were associated with discontinuities of the skin (40%), which were mostly rake marks (for a better appreciation see Supplementary Table S2).

**Table 1:** Macroscopical classification of skin lesions from the present study with their corresponding gross findings.

Category		Description	Gross-findings	Incidence	
				Lesions (*n* = 55)	Percentage (%)
1. Tattoo-like	a. Oval-shaped	Round to irregular well-marked lesions with dark margins and stippled pattern in the centre.	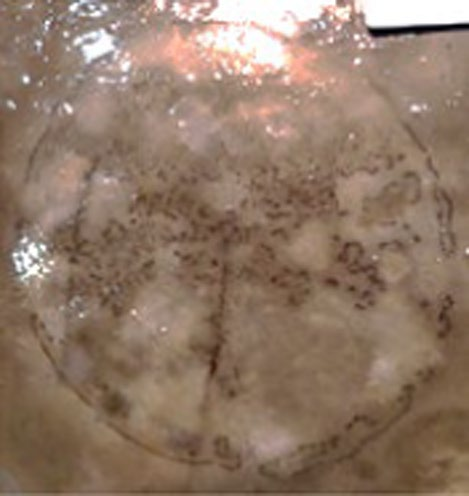 Case 16 (CET 995) *Grampus macrorhynchus* Lesion A1	12	21.81
	b. Coalesced (49)*	Oval-shaped lesions that have coalesced between each other.	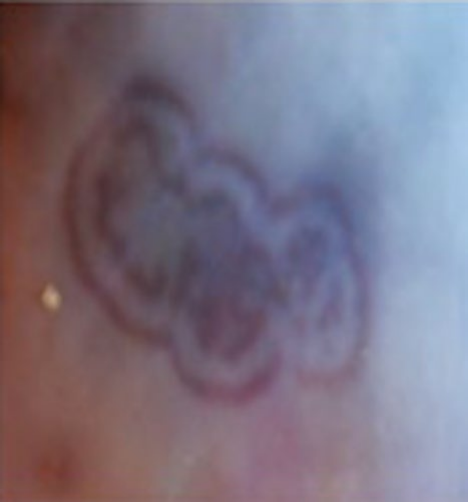 Case 2 (CET 601) *Stenella frontalis* Lesion A1	3	5.45
	c. Serpiginous	Multiple small stippled black lesions very closely located between each other or even coalesced. Their unification and distribution resulted into a serpiginous appearance.	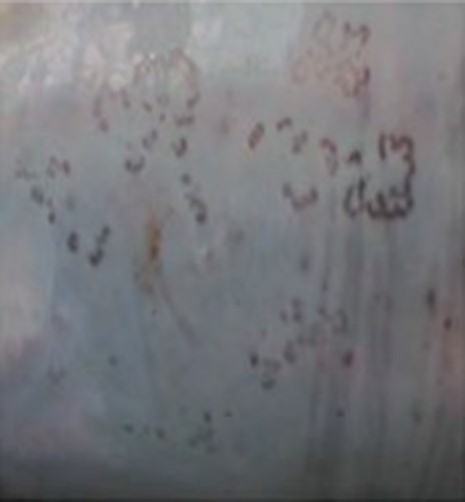 Case 3 (CET 642) *Stenella frontalis* Lesion A1	5	9.09
2. Black-fringed (19)*		This category refers to those round lighter patches in contrast to the average coloration of the skin, with blurred black margins. Occasionally, they presented a slightly dark pinhole or irregular jagged pattern in the centre.	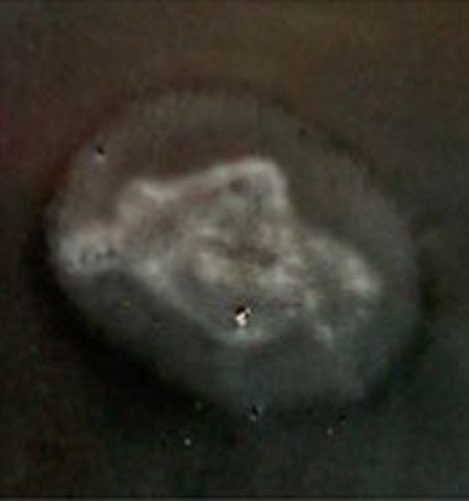 Case 21 (CET 1056) *Stenella frontalis* Lesion A1	9	16.36
3. White-fringed (19)*		This category comprised those round black blemishes or normally colored skin with fade whitish margins. In some cases, an irregular pattern can be present in the centre of the lesions.	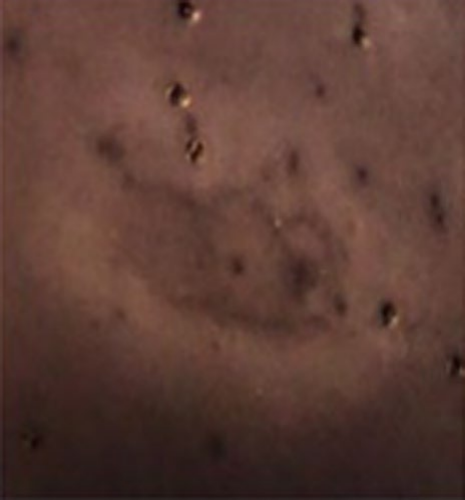 Case 26 (CET 1138) *Stenella frontalis* Lesion A1	8	14.54
4. Pale (19)*		This category refers to pale in color and irregular in shape lesions.	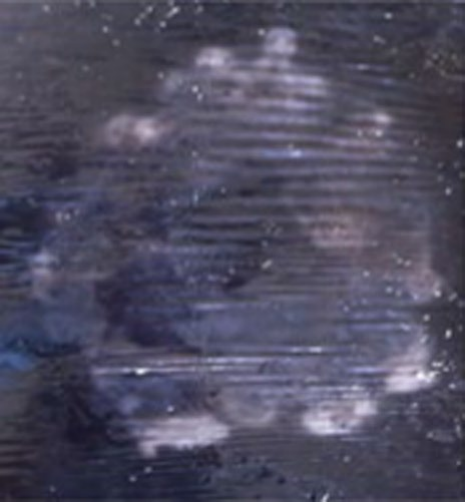 Case 25 (CET 1103) *Tursiops truncatus* Lesion A2	1	1.81
5. Ulcerative (11)*		Irregular shaped open skin lesions with completely loss of the epidermis.	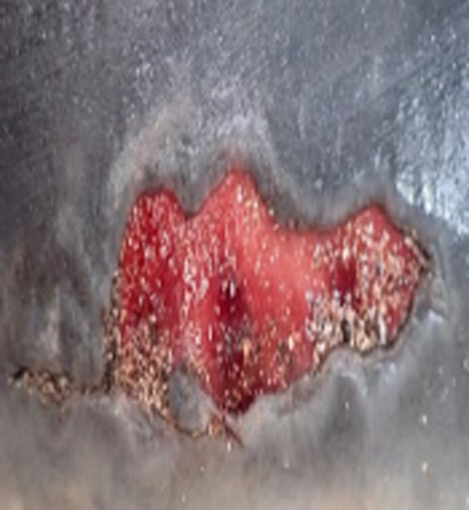 Case 20 (CET 1045) *Delphinus delphis* Lesion A4	4	7.27
6. Target-like (16)*		This category presented oval lesions with dark margins and depressed centre that occasionally could be eroded or ulcerated.	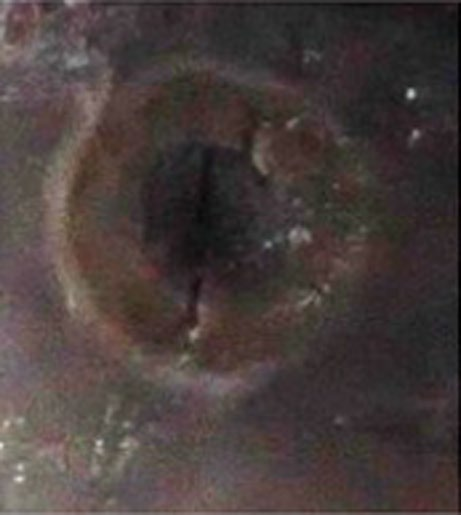 Case 26 (CET 1138) *Stenella frontalis* Lesion A4	3	5.45
7. Ring (11)*		Included in this category were oval flat lesions with uniform divergent colors from black, grey, to white, and even almost imperceptible blemishes that have acquired the color of the normal skin.	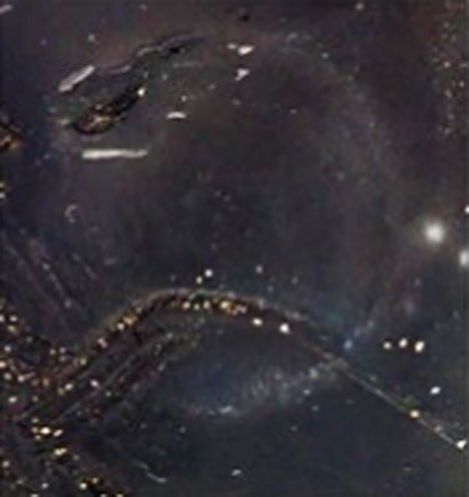 Case 28 (CET 1152) *Stenella frontalis* Lesion A1	6	10.90
8. Tortuous		This category refers to black or white linear lesions setting out tortuous tracts. Additionally, they can show depressed or raised pattern.	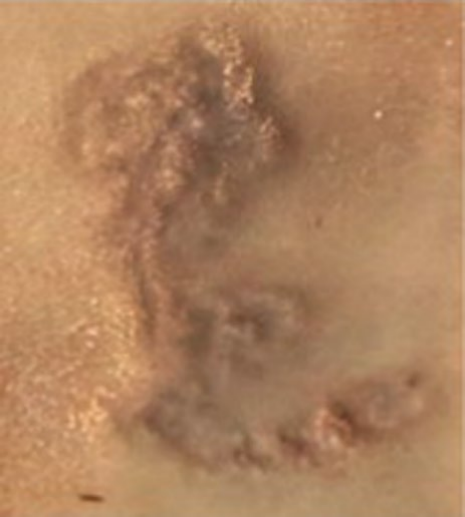 Case 23 (CET 1067); Lesion A3	4	7.27

*N, number of lesions. Asterisks indicate references from which these categories have been previously established.

### 3.2. Molecular findings of skin lesions

Of the 55 skin lesions, 46 were positive (83.63%) for one or more of the selected viruses, and nine were negative (16.36%; see Supplementary Figure S1). CePV-1 was exclusively detected in 21 (38.18%) of the skin lesions, HV was present in 15 (27.27%; 13 were positive for alphaherpesvirus and two for gammaherpesvirus), and evidence of CeMV was found in only one (1.82%). CePV-1 and HV coinfection was detected in nine of the 55 skin lesions (16.36%; see Table 2).

**Table 2:** Molecular results from the 55 skin lesions of the 31 animals stranded on Canary coasts between 2011 and 2021 tested on the present study.

Case N.	ID code	Species	Lesion	MC	PCR results			CT values			Sequences		
					CePV-1	HV	CeMV	CePV-1	HV	CeMV	CePV-1	HV	CeMV
					*N* = 21/55	*N* = 15/55	*N* = 1/55						
1	CET 566	*S. coeruleoalba*	A1	WF	−	−	−	−	−	−	−	−	−
2	CET 601	*S. frontalis*	A1	TL-C	+	−	−	22.89	−	−	ON600451	−	−
3	CET 642	*S. frontalis*	A1	TL-S	**+**	**+**	**−**	**19.24**	**27.19**	**−**	**ON600452**	**OM456331**	**−**
4	CET 663	*D. delphis*	A1	TL-O	+	−	−	20.95	−	−	ON600453	−	−
5	CET 705	*S. coeruleoalba*	A1	TL-S	+	−	−	22.09	−	−	ON600454	−	−
6	CET 748	*S. coeruleoalba*	A1	TL-S	+	−	−	32.10	−	−	ON600455	−	−
7	CET 751	*G. griseus*	A1	T-LO	+	−	−	20.06	−	−	ON600456	−	−
8	CET 947	*D. delphis*	A3	TL-O	**+**	**+**	**−**	**17.73**	**35.64**	**−**	**ON600460**	**OM456332**	**−**
9	CET 951	*S. coeruleoalba*	A1	TL-O	**+**	**+**	**−**	**34.83**	**24.17**	**−**	**ON600461**	**OM456333**	**−**
10	CET 959	*S. coeruleoalba*	A1	U	−	−	−	−	−	−	−	−	−
11	CET 969	*G. macrorhynchus*	A6	TL-O	+	−	−	34.72	−	−	ON600462	−	−
12	CET 983	*S. coeruleoalba*	A3	TL-C	+	−	−	35.13	−	−	ON600463	−	−
13	CET 984	*G. griseus*	A4	TL-O	**+**	**+**	**−**	**36.05**	**36.83**	**−**	**ON600464**	**OM456334**	**−**
14	CET 985	*S. coeruleoalba*	A1	TL-S	**+**	**+**	**−**	**37.49**	**34.79**	**−**	**ON600465**	**OM456335**	**−**
15	CET 991	*S. coeruleoalba*	A3	R	−	−	−	−	−	−	−	−	−
16	CET 995	*G. macrorhynchus*	A1	TL-O	+	−	−	22.04	38.20	−	ON600457	−	−
17	CET 1020	*T. truncatus*	A1	TL-O	**+**	**+**	**−**	**13.79**	**35.55**	**−**	**ON600466**	**OM456336**	**−**
18	CET 1035	*S. coeruleoalba*	A2	BF	−	−	+	−	−	22.32	−	−	**ON314830**
19	CET 1044	*S. frontalis*	A1	R	−	+	−	−	29.21	−	−	OM456337	−
20	CET 1045	*D. delphis*	A4	U	−	+	−	−	37.60	−	−	OM456338	−
21	CET 1056	*S. frontalis*	A1	BF	−	+	−	−	24.75	−	−	OM456339	−
22	CET 1058	*S. frontalis*	A1	BF	**+**	**+**	**−**	**38.41**	**33.86**	**−**	**ON600467**	**OM456340**	**−**
23	CET 1067	*S. frontalis*	A3	Ts	−	+	−	36.30	31.70	−	−	ON314829	−
24	CET 1069	*S. coeruleoalba*	A1	R	+	−	−	37.21	−	−	ON600468	−	−
25	CET 1103	*T. truncatus*	A2	P	−	+	−	−	35.31	−	−	OM456341	−
26	CET 1138		A1	WF	−	+	−	−	19.27	−	−	OM456342	−
			A2	WF	−	+	−	−	23.90	−	−	OM456342	−
		*S. frontalis*	A3	T	−	+	−	−	35.80	−	−	OM456342	−
			A4	T	−	−	−	−	−	−	−	−	−
			A5	U	−	+	−	−	21.22	−	−	OM456342	−
27	CET 1151	*T. truncatus*	A1	TL-S	**+**	**+**	**−**	**23.65**	**32.60**	**−**	**ON600458**	**OM456343**	**−**
			A3	T	−	+	−	−	−	−	−	OM456344	−
			A4	U	−	+	−	−	29.87	−	−	OM456343	−
			A6	R	**+**	**+**	**−**	**31.80**	**28.52**	**−**	**ON600458**	**OM456344**	**−**
28	CET 1152		A1	Ts	−	+	−	−	36.71	−	−	OM456345	−
			A2	WF	−	+	−	−	21.37	−	−	OM456345	−
		*S. frontalis*	A3	Ts	−	−		−	−	−	−	−	−
			A4	R	−	+	−	−	37.34	−	−	OM456345	−
			A5	BF	−	+	−	−	34.68	−	−	OM456345	−
29	CET 1153	*D. delphis*	A1	BF	−	−	−	−	−	−	−	−	−
			A2	Ts	−	−	−	−	−	−	−	−	−
			A3	BF	−	−	−	−	−	−	−	−	−
30	CET 1173	*S. frontalis*	A1	TL-O	+	−	−	15.65	−	−	ON600459	−	−
			A2	TL-O	+	−	−	18.08	−	−	ON600459	−	−
			A3	TL-O	+	−	−	16.42	−	−	ON600459	−	−
			A4	BF	+	−	−	33.63	−	−	ON600459	−	−
			A5	BF	+	−	−	25.02	−	−	ON600459	−	−
			A6	WF	+	−	−	33.44	−	−	ON600459	−	−
			A7	WF	+	−	−	31.79	−	−	ON600459	−	−
			A8	R	+	−	−	35.37	−	−	ON600459	−	−
			A9	WF	+	−	−	28.49	−	−	ON600459	−	−
			A10	WF	+	−	−	12.01	−	−	ON600459	−	−
31	CET 1181		A1	TL-O	+	−	−	13.11	−	−	ON600469	−	−
		*G. macrorhynchus*	A2	TL-C	−	−	−	−	−	−	−	−	−
			A3	BF	+	−	−	27.43	−	−	ON600469	−	−

*CT, cycle threshold; CePV-1, cetacean poxvirus; CeMV, cetacean morbillivirus; HV, herpesvirus; MC, macroscopic classification; A1–A10 = skin lesion samples 1–10; BF, black-fringed; R, ring; T, target-like; Ts, tortuous; TL-C, tattoo-like, coalesced; TL-S, tattoo-like, serpiginous; TL-O, tattoo-like, oval-shaped; P, pale; U, ulcerative; WF, white-fringed; −, negative. CePV-1 and HV coinfected samples are indicated in boldface.

Overall, 11 of the 31 cetaceans tested exclusively positive for CePV-1 (35.48%); eight were solely positive for HV (25.80%), and CeMV was detected in only one (1.82%). Both HV and CePV-1 viruses were simultaneously detected in eight animals (25.80%). Among these, CET 1151 presented with four lesions, of which two were coinfected. Three cetaceans tested negative for the selected pathogens (9.67%).

A range of Ct values (12.01–38.41) were observed for lesions testing positive for CePV-1 by sq-PCR. For HV-positive lesions, Ct values also ranged widely (19.27–37.60). Generally, coinfected lesions had high Ct values, which were not too divergent for both pathogens.

All macroscopic skin categories were positive for one or more of the selected pathogens (see Table 2). The highest number of positive lesions (whether CePV-1 and/or HV positive) was for the TL-O (*N* = 12). None of these lesions tested negative, which was also true of TL-S lesions (*N* = 5). Seven lesions categorized as WF and BF tested positive for selected pathogens. The remaining macroscopic categories tested positive at similar rates, apart from category P which had only one lesion (which tested positive). All gross categories had similar numbers of negative lesions (one or two).

CePV-1 was present in every subcategory of tattoo-like lesions, as well as in WF, BF, and R lesions. Aside from TL-C lesions, HV was detected in all the remaining macroscopic categories. CeMV was detected in a BF lesion. CePV-1 and HV coinfection was mostly detected in tattoo-like lesions (TL-O and TL-S; *N* = 7).

### 3.3. Phylogenetic findings

**Figure 1:**
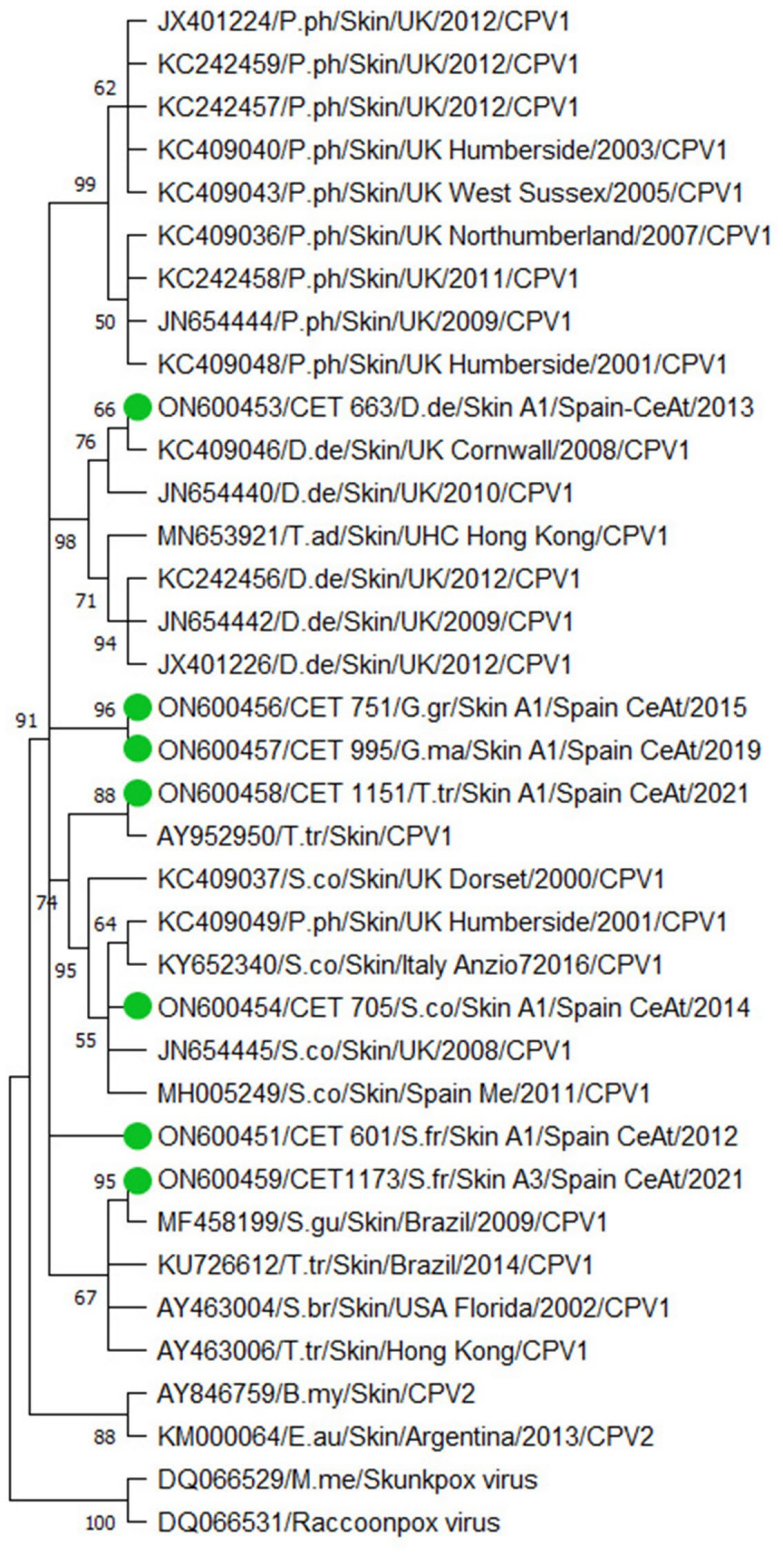
Phylogenetic analysis based on 29 nucleotide sequences from the polymerase gene of cetacean poxvirus. Seven sequences obtained from this study are denoted in colored green circles. The accession number, the identification number, the host, the geographic stranding, and the date of collection were used to identify the nucleotide sequences. B.my (*Balaena mysticetus*); D.de (*Delphinus delphis*); E.au (*Eubalaena australis*); G.gr (*Grampus griseus*); G.ma (*Globicephala macrorhynchus*); M.me (*Mephitis mephitis*); P.ph (*Phocoena phocoena*); S.br (*Steno bredanensis*); S.co (*Stenella coeruleoalba*); S.fr (*Stenella frontalis*); T.ad (*Tursiops aduncus*); T.tr (Tursiops truncates) CeAt (Central Atlantic Ocean); Me (Mediterranean Sea). To construct the tree, we designed the Neighbor-Join and BioNJ algorithms along with the Tamura 3-parameter model and Gamma distribution to model the evolutionary rate differences among sites [five categories (+G, parameter = 0.5213)]. The Bootstrap method was performed to resample 500 replicates and evaluate the reliability of the tree.

In this study, 36 sequences were obtained: 19 CePV-1 and 16 HV sequences based on the polymerase genes, and one CeMV phosphoprotein gene sequence (summarized in Table 2). Nine CePV-1 DNA polymerase products (353–524 bp) and ten other amplicons with shorter lengths (77–99 bp) were obtained. Figure 1 shows the corresponding phylogenetic tree in which only longer sequences and dereplicated sequences were considered. The phylogenetic tree was formed from seven amplicons along with 25 CePV-1 and two CePV-2 GenBank sequences, with the addition of two outgroup sequences (a skunkpox virus and a raccoonpox virus). The sequence obtained from the common dolphin (ON600453) clustered together (bootstrap value (BV) of 98%) with five sequences from common dolphins stranded in the United Kingdom and one Indo-Pacific bottlenose dolphin. Two CePV-1 sequences from a Risso’s dolphin (ON600456) and a short-finned pilot whale (ON600457) of our study were clustered together (BV of 96%). The sequence of the common bottlenose dolphin (ON600458) was grouped (BV of 88%) with a sequence detected in another animal of the same species. The sequence of the striped dolphin (ON600454) was in the same cluster (BV of 95%) with four other sequences from striped dolphins from the United Kingdom and Italy and one harbor porpoise stranded in the United Kingdom. Regarding the sequences obtained of the Atlantic spotted dolphin species in our study, one of them (ON600451) did not cluster with any other sequences of the phylogenetic tree, while the other (ON600459) clustered (BV of 95%) with a sequence obtained from a Guiana dolphin stranded in Brazil.

**Figure 2:**
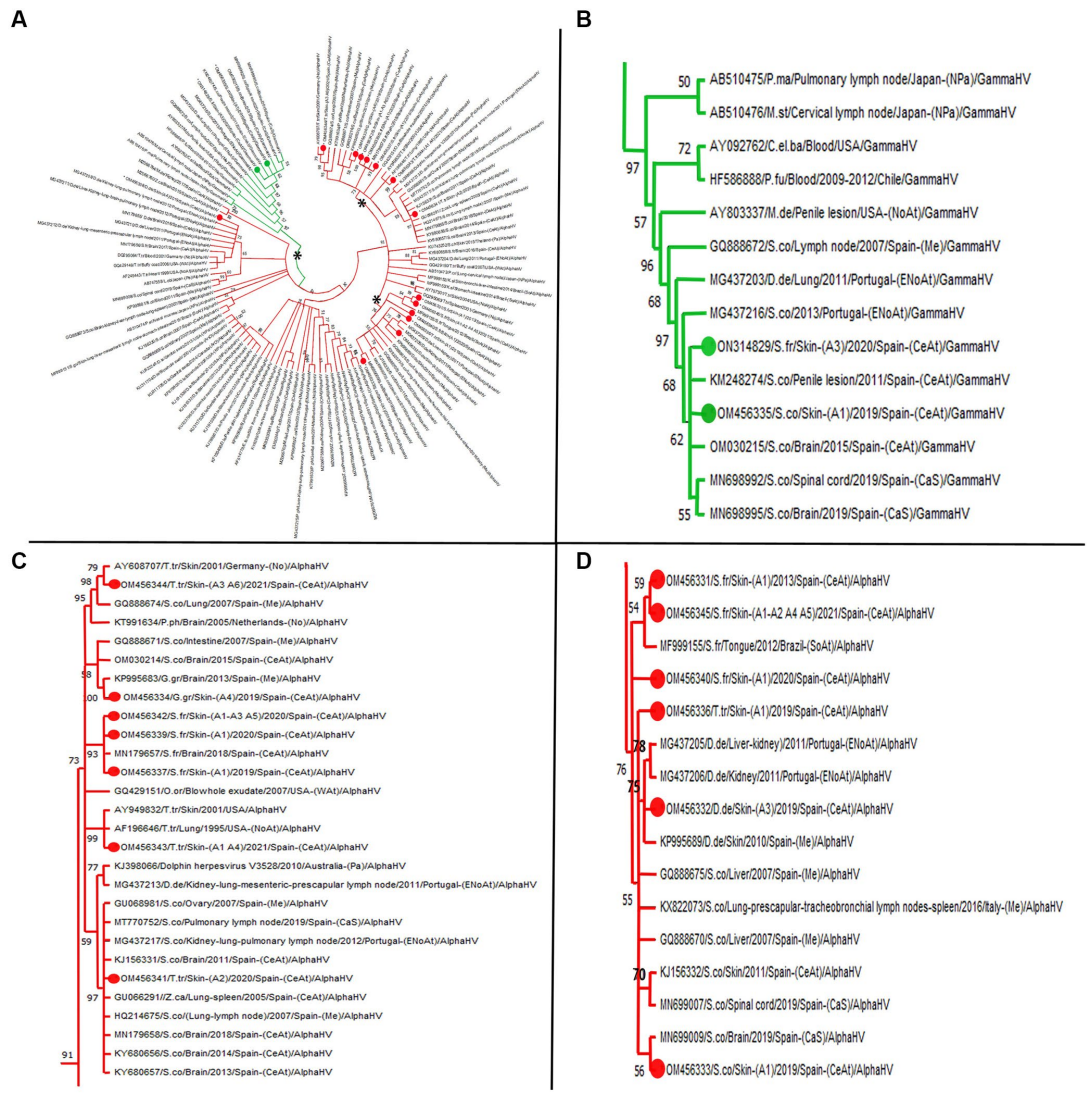
Maximum likelihood phylogenetic tree. **(A)** Molecular phylogenetic analysis based on 91 nucleotide sequences from the polymerase gene of cetacean herpesvirus. 16 sequences obtained from this study are denoted in green (gammaherpesvirus) and red (alphaherpesvirus) colored circles. The accession number, the identification number, the host, the geographic stranding, and the date of collection were used to identify the nucleotide sequence. Asterisks remarks representative clusters. **(B)** Clade with 14 GenBank available cetacean gammaherpesvirus sequences among which two were obtained in the present study. **(C,D)** Clades with different bootstrap values grouping most representative alphaherpesvirus sequences obtained. **(C)** Remark sequence with GenBank acc.no. OM456341 obtained from case 25 (skin lesion A2) which shows a 97% similarity with sequences obtained from other tissues rather than skin. **(D)** Note the big clade with bootstrap value of 76, grouping sequences in several subclades according to species. C.el.ba (*Cervus elaphus barbarous*); D.de (*Delphinus delphis*): G.gr (*Grampus griseus*); M.de (*Mesoplodon densirostris*); M.st (*Mesoplodon stejnegeri*); P.fu (*Pseudalopex fulvipes*); S.co (*Stenella coeruleoalba*); S.fr (*Stenella frontalis*); T.tr (*Tursiops truncates*); Z.ca (*Ziphius cavirostris*); P.ma (*Physeter catodon*); P.ph (*Phocaena phocaena*); NoAt (North Atlantic Ocean); ENoAt (Northeast Atlantic Ocean); WAt (West Atlantic Ocean); CeAt (Central Atlantic Ocean); SoAt (South Atlantic Ocean); Me (Mediterranean Sea); CaS (Cantabrian Sea); Pa (Pacific Ocean); NPa (North Pacific Ocean); No (North Sea); ArO (Arctic Ocean). To construct the tree, we designed the Neighbor-Join and BioNJ algorithms along with the Tamura 3-parameter model and Gamma distribution to model the evolutionary rate differences among sites (five categories (+G, parameter = 0.5319)). The Bootstrap method was performed to resample 1,000 replicates and evaluate the reliability of the tree.

Amplicons (*n* = 16) with 193, 191, 190, 181, and 169 bp were identified from the 24 skin lesions that tested positive for HV (Supplementary Figure S2). Three large clusters (one for *gammaherpesvirus* and two for alphaherpesvirus sequences arising from the same branch supported by a BV of 91%) comprising several of the HV sequences were identified in the phylogenetic tree (Figure 2A). Gammaherpesvirus sequences (*n* = 2) were clustered together among other sequences from the same herpesvirus subfamily with a relation of 97% (Figure 2B). Remarkably, both sequences were closely related to a virus detected in a penis lesion of a striped dolphin stranded in the same geographic area (GenBank KM248274). Regarding alphaherpesvirus sequences, one large cluster (BV 73%; Figure 2C) contained seven sequences from our study, with three obtained from the common bottlenose dolphin, three from the Atlantic spotted dolphin, and one from the Risso’s dolphin species. All sequences, except one from a common bottlenose dolphin (OM454361), were in well-supported groups with other sequences obtained from animals of the same species, with BVs > 70%. Concerning sequence OM454361, it was clustered with a BV of 97% with sequences detected in several cetacean species that shared some characteristics, including necrosis and the presence of intranuclear inclusion bodies (INIB) in the affected organs. The other large cluster within the *Alphaherpesvirinae* subfamily was supported by a BV of 76% (Figure 2D) and contained six sequences: three were from the Atlantic spotted dolphin. The sequence from the common dolphin was grouped (BV of 75%) with sequences detected in animals of the same species stranded along the coasts of Portugal and Spain. Finally, a sequence detected in a common dolphin in our study (OM454338) was in a separate branch (BV of 97%) in which there are no other sequences detected in the skin. This sequence clustered (BV of 73%) with sequences detected in common dolphins stranded in Portugal and the Canary Islands, an Atlantic spotted dolphin stranded in the Canary Islands, and common bottlenose dolphins stranded in the United States and Germany (Figure 2A). Supplementary Table S3 reveals more concisely the percent identity of each study sequence with the closest GenBank match.

Lastly, sequencing of the P gene fragment of the product obtained from CET 1035 (167 bp) revealed a relation of 100% with DMV detected in the lung of a fin whale (*Balaenoptera physalus*) stranded in Denmark in 2016 (GenBank MH430939), in a Risso’s dolphin stranded in the Canary Islands in 2015 (GenBank KY886370) and in a bottlenose dolphin stranded in the United States in 2013 (GenBank KU720622). Additionally, this similarity was observed for sequences derived from the lung, brain, pulmonary and mesenteric lymph nodes, spleen, kidney, and liver samples from striped dolphins stranded in Galicia and Portugal waters.

### 3.4. Histopathological and immunochemical findings

**Figure 3:**
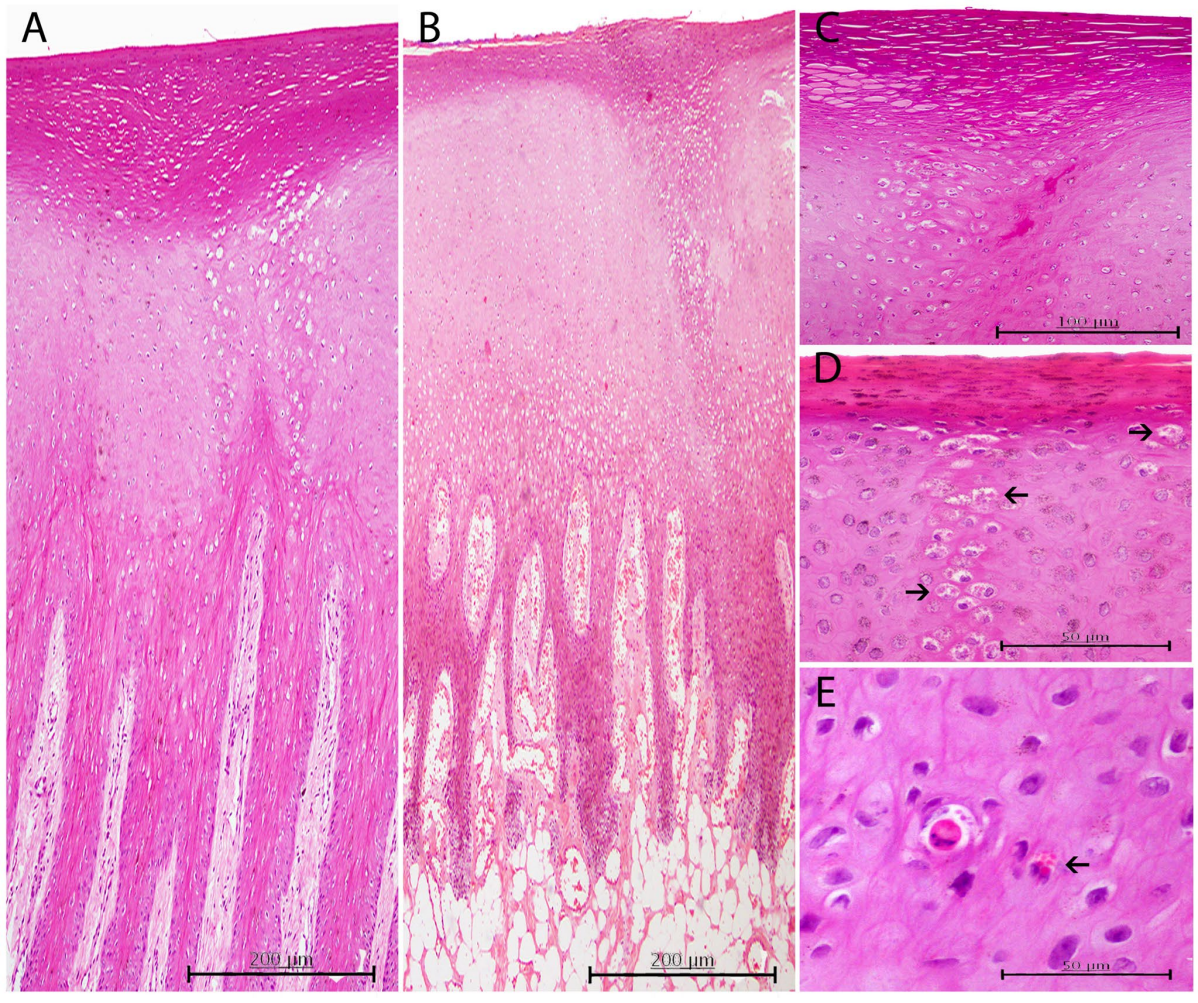
Histopathological findings in CePV-1 positive skin lesions from five cases. **(A)** Lesion A1 from case 7. Focal marked hyperkeratosis showing two focal columns of ballooning degeneration affecting apical areas of rete ridges and the epidermal transitional zone between both stratums corneum and spinosum. H and E, ×10. **(B)** Lesion A1 from case 16. Focal zone of moderate ballooning degeneration affecting both stratum corneum and spinosum. Marked hyperkeratosis just above the line of vacuolated keratinocytes is observed. Marked multifocal congestion in the dermal papillae. H and E, ×10. **(C)** Lesion A6 from case 11. Marked focal hyperkeratosis. Beneath this affected area, a moderate focal ballooning degeneration in the stratum spinosum is appreciated. H and E, ×20. **(D)** Lesion A1 from case 30. ICIBs detected in a column-like group of vacuolized keratinocytes (arrows). Right above, mild hyperkeratosis with associated slightly hyperpigmented keratinocytes. HE, ×40. **(E)** Lesion A1 from case 31. Acidophilic apoptotic keratinocyte with small amphophilic ICIBs. Multiple irregular sized ICIBs in a vacuolated keratinocyte (arrow). H and E, ×40.

Thirty-eight of the 55 skin samples were considered adequate for histopathological examination (69.1%; Supplementary Table S4). Based on the analysis of the most prevalent microscopic findings and etiologies (Table 3), acanthosis (68.16%) and ballooning degeneration (54.53%) were considered the predominant histopathological changes in skin lesions positive for CePV-1. Vacuolized epidermal cells were multifocally concentrated in apical areas of this layer or created linear columns (Figure 3A), which rarely expanded laterally to create multifocal cones (Figure 3B). Where ballooning degeneration was observed, simultaneous moderate multifocal hyperkeratosis was typically observed (59.09%; Figure 3C), which in turn was associated with mild focal hyperpigmentation (31.81%). More rarely (27.27%), small, round, irregular, and pale eosinophilic intracytoplasmic inclusion bodies (ICIBs) were observed in vacuolized keratinocytes (Figures 3D,E).

**Table 3:** Percentages and number of lesions presenting each histopathological finding grouped by etiologies.

Skin associated lesions		CePV-1 (*n* = 22)		HV (*n* = 15)		CeMV (*n* = 1)		Coinfection (*n* = 9)	
		Lesions (*n*)	Percentage (%)	Lesions (*n*)	Percentage (%)	Lesions (*n*)	Percentage (%)	Lesions (*n*)	Percentage (%)
Hyperkeratosis	Minimal	4	18.18	2	13.33	1	100	0	0
	Mild	4	18.18	3	20	0	0	3	33.33
	Moderate	5	22.72	1	6.67	0	0	2	22.22
	Severe	0	0	0	0	0	0	0	0
		**13**	**59.09**	**6**	**40**	**1**	**100**	**5**	**55.55**
Acanthosis	Minimal	7	31.81	1	6.66	0	0	0	0
	Mild	7	31.81	3	20	1	100	5	55.55
	Moderate	1	4.54	2	13.33	0	0	1	11.11
	Severe	0	0	0	0	0	0	0	0
		**15**	**68.16**	**6**	**39.99**	**1**	**100**	**6**	**66.66**
Ballooning degeneration	Minimal	6	27.27	0	0	0	0	1	11.11
	Mild	2	9.09	0	0	0	0	3	33.33
	Moderate	3	13.63	0	0	0	0	2	22.22
	Severe	1	4.54	0	0	0	0	0	0
		**12**	**54.53**	**0**	**0**	**0**	**0**	**6**	**66.66**
Spongiosis	Minimal	2	9.09	0	0	0	0	1	11.11
	Mild	1	4.54	0	0	0	0	0	0
	Moderate	0	0	0	0	0	0	0	0
	Severe	4	18.18	0	0	0	0	0	0
		**7**	**31.81**	**0**	**0**	**0**	**0**	**1**	**11.11**
Necrosis	Minimal	0	0	3	20	0	0	2	22.22
	Mild	0	0	1	6.66	0	0	0	0
	Moderate	0	0	1	6.66	0	0	1	11.11
	Severe	0	0	0	0	0	0	0	0
		**0**	**0**	**5**	**33.32**	**0**	**0**	**3**	**33.33**
Satellitosis		**0**	**0**	**1**	**6.67**	**0**	**0**	**1**	**6.67**
Hyperpigmentation	Minimal	4	18.18	1	6.66	0	0	0	0
	Mild	3	13.63	0	0	0	0	0	0
	Moderate	0	0	0	0	0	0	0	0
	Severe	0	0	0	0	0	0	0	0
		**7**	**31.81**	**1**	**6.66**	**0**	**0**	**0**	**0**
Hypopigmentation	Minimal	0	0	1	6.66	0	0	0	0
	Mild	0	0	2	13.33	0	0	0	0
	Moderate	0	0	0	0	0	0	0	0
	Severe	0	0	0	0	0	0	0	0
		**0**	**0**	**3**	**20**	**0**	**0**	**0**	**0**
Fused rete ridges	Minimal	2	9.09	2	13.33	0	0	2	22.22
	Mild	0	0	0	0	0	0	0	0
	Moderate	0	0	1	6.66	1	1	0	0
	Severe	0	0	1	6.66	0	0	0	0
		**2**	**9.09**	**4**	**26.65**	**1**	**100**	**2**	**22.22**
ICIBs		**6**	**27.27**	**0**	**0**	**0**	**0**	**6**	**66.66**
INIBs		**0**	**0**	**1**	**6.66**	**0**	**0**	**1**	**6.67**
Inflammatory cell infiltration	Minimal	8	36.36	3	20	1	100	2	22.22
	Mild	4	18.18	0	0	0	0	3	33.33
	Moderate	1	4.54	0	0	0	0	0	0
	Severe	0	0	3	20	0	0	0	0
		**13**	**59.09**	**6**	**40**	**1**	**100**	**5**	**55.55**
Congestion	Minimal	4	18.18	2	13.33	0	0	1	6.67
	Mild	2	9.09	2	13.33	0	0	2	22.22
	Moderate	1	4.54	0	0	0	0	2	22.22
	Severe	0	0	1	6.66	0	0	0	0
		**7**	**31.81**	**5**	**33.32**	**0**	**0**	**5**	**51.11**
Dyskeratosis/apoptosis	Minimal	4	18.18	1	6.66	0	0	0	0
	Mild	1	4.54	0	0	0	0	1	6.67
	Moderate	0	0	0	0	0	0	1	6.67
	Severe	0	0	0	0	0	0	0	0
		**5**	**22.72**	**1**	**6.66**	**0**	**0**	**2**	**13.34**
Pearl corns	Minimal	0	0	2	13.33	0	0	2	22.22
	Mild	0	0	1	6.66	0	0	0	0
	Moderate	0	0	0	0	0	0	0	0
	Severe	0	0	0	0	0	0	0	0
		**0**	**0**	**3**	**20**	**0**	**0**	**2**	**22.22**

*CePV-1, cetacean poxvirus; CeMV, cetacean morbillivirus; HV, herpesvirus. Bold indicates the percentages, and underlined numbers represents the highest values.

**Figure 4:**
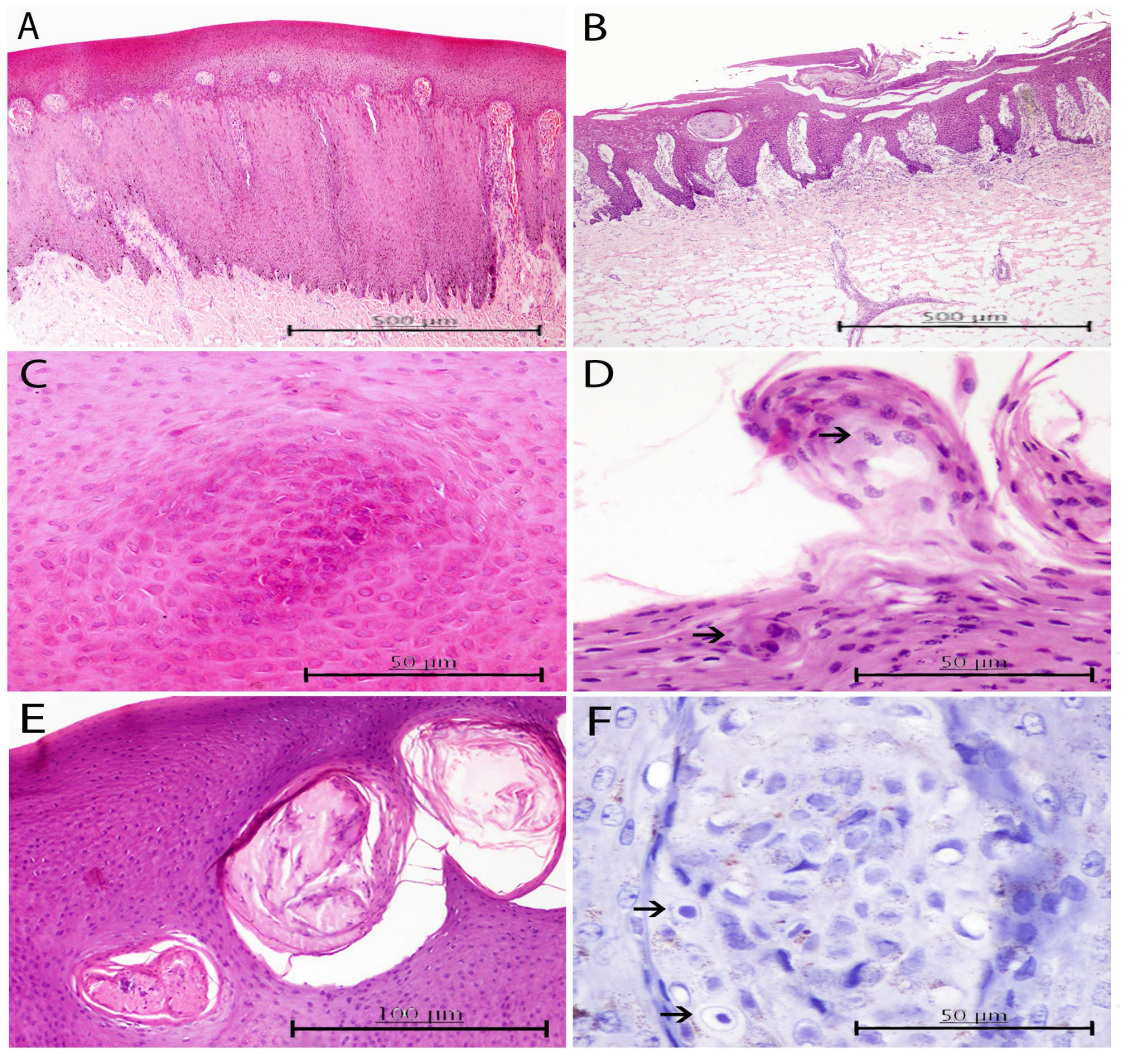
Histopathological findings in HV positive skin lesions from three animals of the present study. **(A)** Lesion A2 from case 25. Moderate to marked hyperkeratosis and acanthosis with elongated fused rete ridges that penetrate down to the dermis. Multifocally, some dermal papillae have been occluded due to anastomosing rete ridges, and congestion is observed in the ones remaining uncapped. H and E, ×4. **(B)** Lesion A3 from case 23. Loss of stratum corneum and part of stratum spinosum with the presence of necrotic cellular crusts. H and E, ×4. **(C)** Detailed image of a focal arrangement of acidophilic keratinocytes with ground glass eosinophilic nuclei in stratum spinosum of the same skin lesion. H and E, ×40. **(D)** Lesion A3 from case 23. Round abnormal keratinocytes with condensed nuclei scattered within the upper areas of the stratum (upper arrow). Focal oval-shaped syncytia of basophilic keratinocytes within the intermediate layer (lower arrow). H and E, ×40. **(E)** Lesion A3 from case 28. Multifocal well-delimited oval necrotic areas containing degenerated keratinocytes and neutrophils within the stratum spinosum. H and E, ×20. **(F)** Lesion A2 from case 25. Evidence of INIBs in the most superficial area of a dermal papillae (arrows). Immunochemistry stain. Canine distemper virus (CDV) antibody, ×60.

Diffuse hyperkeratosis (40%) with acanthotic epithelium (39.99%) was predominantly found in alphaherpesvirus-positive lesions (Figure 4A). In other cases, the distinctive loss of the stratum corneum and part of the stratum spinosum was observed (Figure 4B). Cellular and nuclear pleomorphisms (Figure 4C), as well as multifocal basophilic syncytial keratinocytes, were observed in the apical areas of the stratum spinosum (Figure 4D). In some lesions (33.33%), the stratum spinosum randomly showed mild, multifocal, well-delimited, oval, necrotic areas concentrated with degenerated keratinocytes and neutrophils (Figure 4E). Severe neutrophilic inflammatory cell infiltration in blood vessels was a common finding (40%), while INIBs were difficult to distinguish in all alphaherpesvirus-positive lesions (6.66%).

Regarding the ICH results, immunostaining for HV was not observed in any of the HV-positive (by PCR) skin lesions even though immunostaining was successful for the positive control. Nevertheless, evidence of INIBs was more definite for CET 1103 after immunolabeling than after HE staining (Figure 4F).

**Figure 5:**
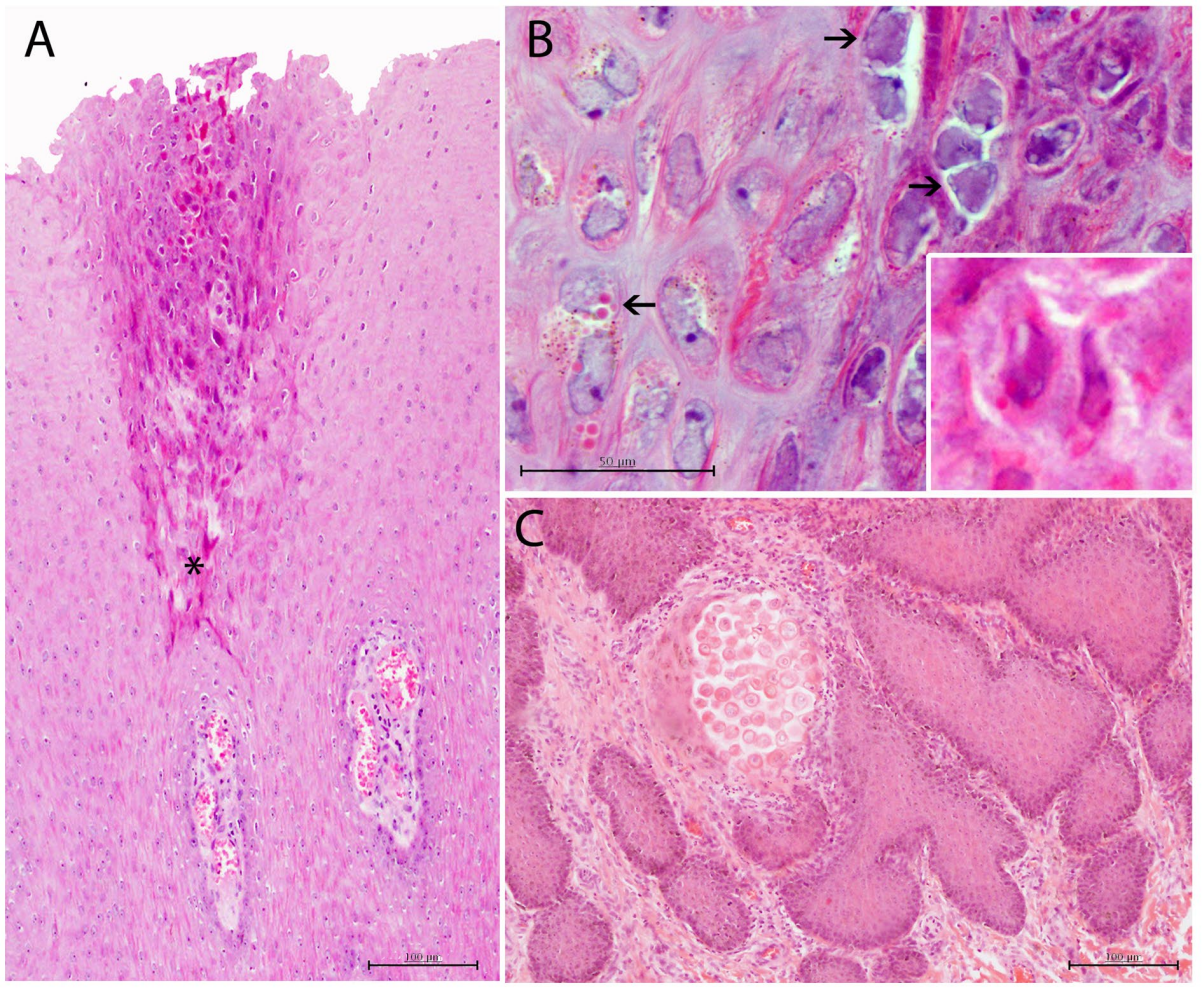
Histopathological findings in CePV-1 and HV coinfected skin lesion from case 9. **(A)** Focal irregular arrangement of acidophilic keratinocytes with both basophilic INIBs and small round amphophilic ICIBs in stratum spinosum. Multifocal mild to moderate ICI in dermal papillae. Asterisk indicates the affected area of the stratum spinosum. H and E, ×20. **(B)** Detail of irregular-shaped keratinocytes with small vacuolizations and prominent basophilic INIBs (right upper arrows) and small round pinpoint amphophilic ICIBs (lower left arrow). Lower inset: zoomed-in image of a keratinocyte with both INIBS and ICIBs. H and E, ×60. **(C)** Focal delimited area with abnormal acidophilic necrotic keratinocytes in the basal area of a dermal papilla associated to a combined neutrophilic and eosinophilic ICI. H and E, ×20.

In coinfected lesions, a combination of the above-described histologic changes from both CePV-1 and HV pathogens were observed. Diffuse acanthosis was a common finding (66.66%) along with multifocal ballooning degeneration (66.66%) with associated hyperkeratosis (55.55%). Almost all coinfected lesions presented ICIBs in which the typical umbrella-like arrangement or “melanin-cap” was noticeably absent. Conversely, INIBs were only noticed in CET 951, where both ICIBs and INIBs were apparent with obvious multifocal syncytial organizations (Figure 5A). Irregular ICIBs (Figure 5B) and multifocal apoptotic-like keratinocytes were observed through the intermediate layer at a mild to moderate degree (13.34%; Figure 5C). Combined mild to moderate lymphocytic and neutrophilic inflammatory cell infiltration and congestion were observed in several lesions (55.55%).

**Figure 6:**
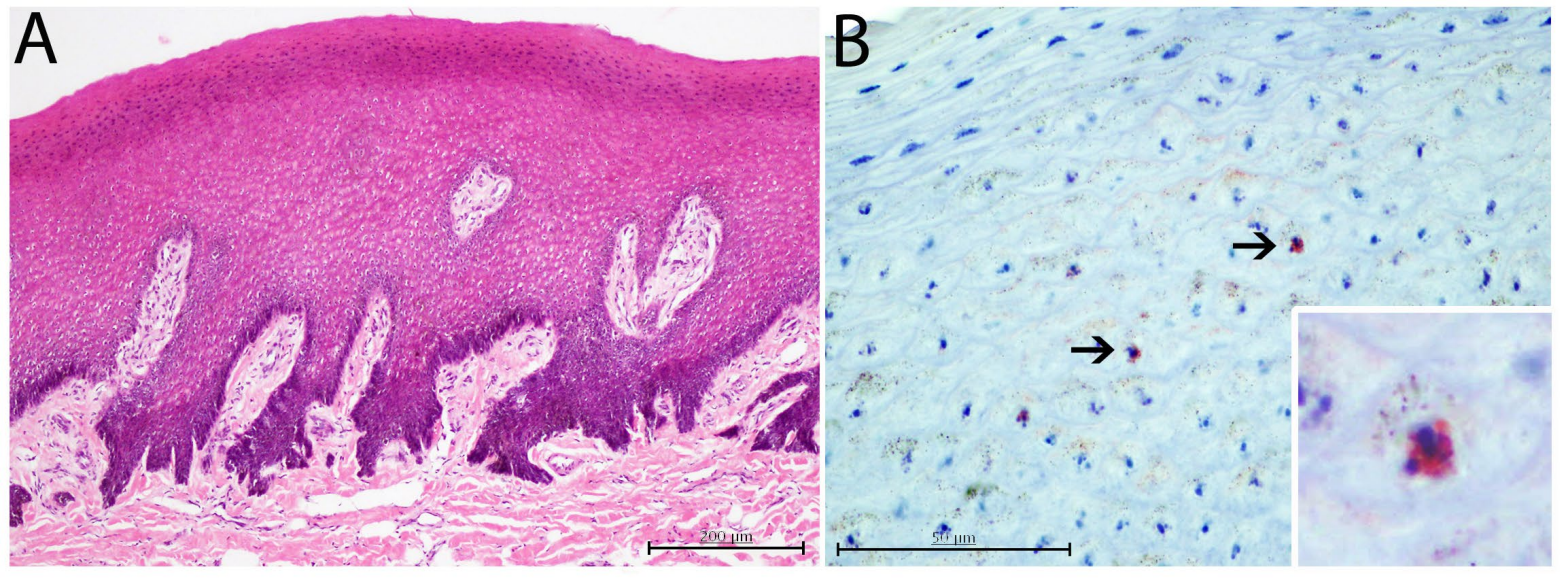
Histopathological and immunohistological findings in CeMV positive skin lesion from case 17. **(A)** Mild to moderate diffuse acanthosis with irregular laterally displaced and fused rete ridges. H and E, ×10. **(B)** Slightly immunostained keratinocytes against canine distemper virus (CDV) antibody. Lower inset: zoomed-in image of an immunostained keratinocyte. Immunochemistry stain, ×60.

The CeMV-positive lesion presented with mild acanthosis with a disorganized histologic architecture for which some rete ridges were laterally fused and almost parallel to the stratum spinosum (Figure 6A). Furthermore, this lesion also tested positive by IHC, with a few random keratinocytes lightly immunolabeled for canine distemper virus (CDV; Figure 6B).

Among pathogen-negative lesions, those from CET 1153 showed moderate diffuse acanthosis. Inflammatory cell infiltration (ICI) was multifocally observed in the apical areas of the dermal papillae. The remaining negative lesions did not show any remarkable histological changes.

The tattoo-like and BF lesions were the most prevalent by microscopy (Table 4), but these histologic changes were mild to moderate. Preliminarily, 75% of the TL-O lesions showed mild acanthosis, followed by mild to moderate hyperkeratosis, ballooning degeneration, and ICIBs (66.66%). All the TL-S lesions presented mild to moderate ballooning degeneration and congestion. A significant proportion of the latter cases (80%) were associated with mild to moderate hyperkeratosis and acanthosis. These two subcategories of tattoo lesions were also among the few in which ICIBs were observed (60%). Of the BF skin lesions, 88.88% showed mild acanthosis, followed by mild to moderate hyperkeratosis (66.66%). A repeated pattern exclusively present in the Ts lesions was observed with well-delimited multifocal areas of degenerative keratinocytes and neutrophils that sometimes merged to the outer layer, leading to mild to moderate disruptions of the stratum corneum. Consequently, 75% of the lesions presented moderate necrosis. Neither ulcered nor target-like lesions are represented in Table 4, as they did not apply to the histologic analysis and/or their histological changes were not evaluable.

**Table 4:** Summary of degree severity of most prevalent histopathological findings by macroscopic categorization of skin lesions of the present study.

		Macroscopic classification of skin lesions															
Skin associated lesions		Tattoo-like oval shaped (*n* = 12)		Tattoo-like coalesced (*n* = 3)		Tattoo-like serpiginous (*n* = 5)		Black-fringed (*n* = 9)		White-fringed (*n* = 8)		Pale (*n* = 1)		Ring (*n* = 6)		Tortuous (*n* = 4)	
		*N*	%	*N*	%	*N*	%	*N*	%	*N*	%	*N*	%	*N*	%	*N*	%
Hyperkeratosis	Minimal	1	8.33	0	0	0	0	2	22.22	2	25	0	0	0	0	1	25
	Mild	3	25	0	0	2	40	2	22.22	0	0	1	100	1	16.66	2	50
	Moderate	4	33.33	0	0	2	40	2	22.22	0	0	0	0	0	0	1	25
	Severe	0	0	0	0	0	0	0	0	0	0	0	0	0	0	0	0
		**8**	**66.66**	**0**	**0**	**4**	**80**	**6**	**66.66**	**2**	**25**	**1**	**100**	**1**	**16.66**	**4**	**100**
Acanthosis	Minimal	1	8.33	1	33.33	0	0	2	22.22	3	37.5	0	0	1	16.66	1	25
	Mild	6	50	0	0	4	80	4	44.44	1	12.5	0	0	1	16.66	3	75
	Moderate	2	16.66	0	0	0	0	2	22.22	0	0	1	100	0	0	0	0
	Severe	0	0	0	0	0	0	0	0	0	0	0	0	0	0	0	0
		**9**	**75**	**1**	**33.33**	**4**	**80**	**8**	**88.88**	**4**	**50**	**1**	**100**	**2**	**33.32**	**4**	**100**
Ballooning degeneration	Minimal	1	8.33	1	33.33	2	40	2	22.22	0	0	0	0	0	0	0	0
	Mild	2	16.66	0	0	2	40	0	0	0	0	0	0	1	16.66	0	0
	Moderate	4	33.33	0	0	1	20	0	0	0	0	0	0	0	0	0	0
	Severe	1	8.33	0	0	0	0	0	0	0	0	0	0	0	0	0	0
		**8**	**66.66**	**1**	**33.33**	**5**	**100**	**2**	**22.22**	**0**	**0**	**0**	**0**	**1**	**16.66**	**0**	**0**
Spongiosis	Minimal	2	16.66	0	0	0	0	0	0	0	0	0	0	0	0	0	0
	Mild	1	8.33	0	0	0	0	1	11.11	0	0	0	0	0	0	0	0
	Moderate	0	0	0	0	0	0	0	0	0	0	0	0	0	0	0	0
	Severe	1	8.33	0	0	0	0	0	0	0	0	0	0	0	0	0	0
		**4**	**33.33**	**0**	**0**	**0**	**0**	**1**	**11.11**	**0**	**0**	**0**	**0**	**0**	**0**	**0**	**0**
Necrosis	Minimal	1	8.33	0	0	2	40	0	0	0	0	1	1	0	0	1	25
	Mild	0	0	0	0	0	0	1	11.11	0	0	0	0	0	0	0	0
	Moderate	1	8.33	0	0	1	20	0	0	0	0	0	0	0	0	2	50
	Severe	0	0	0	0	0	0	0	0	0	0	0	0	0	0	0	0
			**16.66**	**0**	**0**	**3**	**60**	**1**	**11.11**	**0**	**0**	**1**	**100**	**0**	**0**	**3**	**75**
Satellitosis		**1**	**8.33**	**0**	**0**	**0**	**0**	**0**	**0**	**0**	**0**	**0**	**0**	**0**	**0**	**1**	**25**
Hyperpigmentation	Minimal	2	16.66	1	33.33	0	0	0	0	1	12.5	1	100	0	0	0	0
	Mild	2	16.66	0	0	0	0	1	11.11	0	0	0	0	0	0	0	0
	Moderate	0	0	0	0	0	0	0	0	0	0	0	0	0	0	0	0
	Severe	0	0	0	0	0	0	0	0	0	0	0	0	0	0	0	0
		**4**	**33.33**	**1**	**33.33**	**0**	**0**	**1**	**11.11**	**1**	**12.5**	**1**	**100**	**0**	**0**	**0**	**0**
Hypopigmentation	Minimal	0	0	0	0	0	0	0	0	0	0	1	100	0	0	0	0
	Mild	0	0	0	0	0	0	1	11.11	0	0	0	0	0	0	1	25
	Moderate	0	0	0	0	0	0	0	0	0	0	0	0	0	0	0	0
	Severe	0	0	0	0	0	0	0	0	0	0	0	0	0	0	0	0
		**0**	**0**	**0**	**0**	**0**	**0**	**1**	**11.11**	**0**	**0**	**1**	**100**	**0**	**0**	**1**	**25**
Fused rete ridges	Minimal	2	16.66	0	0	0	0	1	11.11	2	25	0	0	0	0	0	0
	Mild	0	0	0	0	0	0	0	0	0	0	0	0	0	0	1	25
	Moderate	0	0	0	0	0	0	3	33.33	0	0	0	0	0	0	1	25
	Severe	0	0	0	0	0	0	0	0	0	0	1	100	0	0	0	0
		**2**	**16.66**	**0**	**0**	**0**	**0**	**4**	**44.44**	**2**	**25**	**1**	**100**	**0**	**0**	**2**	**50**
ICIBs		**8**	**66.66**	**0**	**0**	**3**	**60**	**0**	**0**	**0**	**0**	**0**	**0**	**1**	**16.66**	**0**	**0**
INIBs		**1**	**8.33**	**0**	**0**	0	0	**0**	**0**	**0**	**0**	**1**	**100**	**0**	**0**	**0**	**0**
ICI	Minimal	3	25	1	33.33	1	20	5	55.55	4	50	0	0	1	16.66	2	50
	Mild	4	33.33	0	0	2	40	1	11.11	0	0	0	0	0	0	0	0
	Moderate	0	0	0	0	0	0	2	22.22	0	0	1	100	0	0	2	50
	Severe	0	0	0	0	0	0	0	0	0	0	0	0	0	0	0	0
		**7**	**58.33**	**1**	**33.33**	**3**	**60**	8	**88.88**	**4**	**50**	**1**	**100**	**1**	**16.66**	**4**	**100**
Congestion	Minimal	3	25	0	0	1	20	2	22.22	2	25	0	0	1	16.66	1	25
	Mild	2	16.66	0	0	1	20	2	22.22	1	12.50	1	100	0	0	0	0
	Moderate	0	0	0	0	3	60	0	0	0	0	0	0	0	0	0	0
	Severe	1	8.33	0	0	0	0	0	0	0	0	0	0	0	0	0	0
		**6**	**50**	**0**	**0**	**5**	**100**	**4**	**44.44**	**3**	**37.5**	**1**	**100**	**1**	**16.66**	**1**	**25**
Dyskeratosis/apoptosis	Minimal	2	16.66	0	0	0	0	4	44.44	0	0	0	0	0	0	0	0
	Mild	2	16.66	0	0	0	0	0	0	0	0	0	0	0	0	0	0
	Moderate	1	8.33	0	0	0	0	0	0	0	0	0	0	0	0	0	0
	Severe	0	0	0	0	0	0	0	0	0	0	0	0	0	0	0	0
		**5**	**41.66**	**0**	**0**	**0**	**0**	**4**	**44.44**	**0**	**0**	**0**	**0**	**0**	**0**	**0**	**0**
Pearl corns	Minimal	1	8.33	1	33.33	0	0	0	0	1	12.5	0	0	1	16.66	0	0
	Mild	0	0	0	0	0	0	0	0	0	0	0	0	0	0	1	25
	Moderate	0	0	0	0	0	0	0	0	0	0	0	0	0	0	0	0
	Severe	0	0	0	0	0	0	0	0	0	0	0	0	0	0	0	0
		**1**	**8.33**	**1**	**33.33**	**0**	**0**	**0**	**0**	**0**	**12.5**	**0**	**0**	**1**	**16.66**	**1**	**25**

*ICIBs, intracytoplasmic inclusion bodies; INIBs, intranuclear inclusion bodies; *N*, number of lesions.

Bold numbers indicate n and percentage of main histopathological changes observed in each macroscopical category of skin lesion. Higher numbers and percentages are underlined.

## 4. Discussion

Because of their limited accessibility, most pro-active health studies in free-ranging cetaceans exclusively assess their skin conditions using only visual appraisals for diagnosis (23, 71), which results in a high risk for misinterpretation of skin disease pathogens. Therefore, stranded cetaceans are critical study subjects that provide unlimited access and the opportunity to fully comprehend skin diseases and their impact on the health of marine mammals. Hence, this study represents the first multidisciplinary study involving macroscopic, histological, and molecular analyses of a significant number of viral skin lesions in several species of stranded cetaceans. Molecular identification of CePV in poxvirus-like skin lesions has been performed in several species (20, 51, 72). However, to the authors’ knowledge, the present study is the first to identify this virus in pilot whales. HV infections have been identified in several cetacean species and tissue samples (20, 73, 74). However, HV DNA has not been reported in skin lesions of Risso’s dolphins, which makes the present study the foremost publication on HV related to skin lesions in this species.

Viral skin lesions in these marine mammals are generally considered potential health indicators (14, 75). Most studies have focused on recognizing TSD lesions because of their wide global distribution and characteristic and distinguishable presentations; the molecular identification of CePV has been associated with these lesions (76, 77). However, to the best of our knowledge, no studies have surveyed viral pathogens other than CePV nor their co-occurrence in CePV-positive cetacean skin lesions. Most studies of CePV coinfection have implicated tissues other than the skin; Melero and co-workers (78) detected both poxvirus and HV in the tonsil of a Pacific walrus (*Odobenus rosmarus divergens*). To our knowledge, this investigation is the first to corroborate HV and CePV coinfection in marine mammals; previous studies of concomitant skin lesion infections by both agents have been conducted in other species such as hares (*Lepus*), while leporipoxvirus and leporid gammaherpesvirus-5 co-infections were recently reported (79). In cattle, an outbreak of lumpy skin disease virus and bovine herpesvirus-4 occurred in Egypt where cows showed generalized deep skin nodules among other clinical signs (80). Reports exist of commercial chicken flocks showing wart-like lesions consistent with fowl poxvirus and severe respiratory manifestations from infectious laryngotracheitis virus (81). HV and CeMV coinfection has been detected in multiple organs of a few cetaceans (28, 82, 83), as well as CeMV and *Brucella* sp. in central nervous system (29, 84). Nevertheless, this is the first report revealing a considerable prevalence of poxvirus (35.48%) and herpesvirus (25.80%) skin diseases in stranded cetaceans in the Canary Archipelago, in addition to providing the first molecular description of CePV and HV coinfection in cetacean skin lesions (25.80%).

As reported in prior studies, the lesions were mostly observed on visible body parts, especially on dorsal areas, with the head being the most affected (50, 85). Of the eight macroscopic categorizations of 55 skin lesions, the tattoo-like pattern was the most predominant, especially the TL-O form. Usually, this pattern is identified as an early manifestation of TSD (22, 23). The molecular results of the study indicate that all lesions with this presentation are positive for CePV-1, and the majority have high viral loads. However, three oval tattoo-like lesions presented with low Ct values, possibly because a non-representative sample of the lesion was processed for genomic extraction, or because of genomic degradation of the sample. Alternatively, the CePV-1 viral loads may have been affected by HV, which was detected in two of those three tattoo-like lesions. As previously reported (22, 77), the dominant histological findings of tattoo-like lesions were mild to moderate ballooning degeneration associated with hyperkeratosis and acanthosis. Additionally, other acute histopathological processes were moderate vascular congestion with the migration of lymphocytes. Of the three tattoo-like subcategories, the TL-O form showed moderate acute histopathological changes. Furthermore, in correlating Ct values with the latter microscopic findings, this macroscopic category showed early CePV-1 amplifications, which could indicate that these lesions may be the initial manifestations of TSD. Finally, ICIBs were observed in all cases of TL-O, suggesting viral activity.

CePV-1 was also detected in BF, WF, and R lesions, although less frequently. Macroscopically, these skin manifestations can be attributed to poxvirus infection; previous reports have suggested that tattoo-like lesions progress to darker blemishes (persistent stage), turn whiter (regression stage), and become almost invisible (healing stage) (18, 22). The microscopic findings of tattoo-like lesions were observed for the three categories, noting that for BF lesions these histological changes were milder than for WF and R lesions. ICIBs were absent, except for one skin lesion that was coinfected with HV, indicating a possible CePV-1 reactivation. The mild histopathological changes in these macroscopic categories can indicate advanced stages of lesions. Furthermore, all lesions showed high Ct values, which could suggest low viral loads. Together, these findings suggest that the CePV-1-positive skin manifestations may represent chronic stages of the skin disease, thus corroborating these findings with visual diagnostics.

HV was exclusively detected in most gross categories (except the TL-C lesions) across a wide range of skin manifestations, as has been previously reported with wild cetacean populations (45, 71). Furthermore, consistent with previous studies, we commonly observed epidermal necrosis, atypical keratinocytes with both cell and nucleus pleomorphism, and ICI that predominantly involved neutrophils (86, 87). An association between the most prevalent histologic findings and the macroscopic appearance of HV-positive skin lesions was not observed, except for the Ts lesions. Accordingly, all Ts lesions were disrupted in the stratum corneum with well-delimited multifocal crusts of degenerated keratinocytes and neutrophils. Molecular tests revealed that almost all lesions from which HV was identified had high Ct values indicative of low viral loads, suggesting that the lesions could be in chronic or latent stages, though this might also result from poor sampling or nucleic acid degradation. Furthermore, one case in this study showed histopathological changes that were remarkably similar to changes observed in a previously reported HV-positive skin lesion from an Atlantic bottlenose dolphin (34). Both lesions were slightly raised in the stratum corneum, with swollen and irregularly distributed keratinocytes with intranuclear and intracytoplasmic inclusion bodies. In attempting to associate histological changes with the macroscopic appearance of this lesion, Manire and co-workers described the lesion as a hyperplastic area with hundreds of 1–3-mm small spherical firm papules affecting the rostrum, head, dorsal fin, and flanks (34). The lesion in the present study, however, was macroscopically different; it was a TL-O lesion with an apparent porous consistency localized dorsal to the right eye; CePV-1 was also detected in this lesion.

Six of the nine CePV-1 and HV-coinfected lesions showed tattoo-like patterns. To the best of our knowledge, HV has been detected in various skin manifestations (35, 88), excluding these characteristic lesions that have so far been strictly attributed to CePV, and this study is the first to show HV in tattoo-like lesions. Therefore, the diagnosis of a skin pathogen should therefore use molecular tests to corroborate the results of visual assessments. Histologically, in coinfected lesions, the above-mentioned CePV-1 and HV microscopic findings were more severe, in contrast with lesions from which one of these pathogens was exclusively detected. Molecular tests of coinfected skin lesions often showed variable Ct values, but one of the pathogens usually showed high viral loads. Despite this, the HV-associated microscopic changes were generally more prominent than those associated with CePV-1, which may result from the severe infectiousness of HV in the skin (87, 89). Opportunistic pathogens take advantage of pre-existing wounds as portals of entry (40% of the analyzed lesions in this study derive from rake marks), and a conceivable pathway of infection in coinfected skin lesions could be the initial entry of CePV-1 leading to the reactivation of latent HV (90, 91). Another possible scenario, although less likely, would be CePV-1 infection as an initial step leading to an increased susceptibility to a secondary HV infection.

CeMV has also been detected in skin lesions, which are related to rash, erosive, and ulcerative patterns (40, 83, 92). The presence of CeMV in skin lesions (1.82%) in this study was low. However, the detection of CeMV in a BF lesion, which can macroscopically be attributed to advanced poxvirus-like lesions, demonstrates the necessity of evidence-based studies to verify pathogens in skin disorders. Additionally, definitive CeMV-related skin patterns have not yet been established in cetaceans. The detection of this re-emergent systemically infectious virus in a skin lesion is important for monitoring cetacean populations to forecast possible epizootic outbreaks. Indeed, the animal in this study with a CeMV-positive lesion also presented multiorgan infection by this same virus.

From the seven CePV-1 sequences used for constructing the phylogenetic tree, three (from the common dolphin, common bottlenose dolphin, and striped dolphin) were mainly clustered according to their detection in the same host species, which is in accordance with previous reports that proposed that the CPV-1 group may contain several sub-groups specific for the different families of odontocetes (49). The other four sequences from our study were non-clustered or were grouped with sequences detected in other host species, possibly because these host species have no entries in GenBank (Risso’s dolphin, short-finned pilot whale, and Atlantic spotted dolphin).

On the other hand, the sequences were more widely distributed based on the HV phylogenetic tree, with sequences belonging to both *Gammaherpesvirinae* and *Alphaherpesvirinae* subfamilies. Remarkably, as previously reported (93), herpesviruses seem to be host specific, as most of the sequences in our study were grouped with sequences from the same host species. Only two of the herpesvirus sequences in our study belonged to the *Gammaherpesvirinae* subfamily and showed 100% identity with one detected in a penile lesion in a striped dolphin stranded in the Canary Islands (94). This is consistent with reports of this herpesvirus subfamily, which is more frequently detected in genital and mucosal lesions (95), though it has also been detected in the skin (35, 96).

Only three alphaherpesvirus sequences in this study were grouped with sequences previously obtained from skin lesions (86, 96), while most of them were close to sequences acquired from other tissues including ovary (97), pulmonary lymph node (27, 31), kidney, lung, spleen (26), and brain (30). This suggests that the same strains probably affect tissues other than skin. In this sense, another distinct alphaherpesvirus sequence was detected from the adrenal gland of a bottlenose dolphin (case 27, CET 1151), which in turn presented four skin lesions with two different alphaherpesvirus strains. The amplicon recovered from the adrenal gland showed a 100% similarity to a sequence obtained from the skin of a stranded bottlenose dolphin in Germany (86). Moreover, this amplicon was highly similar to another identified from a skin lesion of the same animal, which suggests that the virus may have been disseminated (25).

Finally, one of the skin lesions from this study that histologically presented large intranuclear inclusion bodies surrounded by a clear halo was similar to sequences from animals with HV-related acute and severe lesions including INIBs, necrotic changes, malacia, and lymphoid depletion. Likewise, Eva Sierra and co-workers (30) identified sequences from four cases presenting with severe acute brain lesions that could lead to death; these sequences clustered with the abovementioned pathogenic HV strains. However, as stated above, caution should be exercised when interpreting these short sequences.

## 5. Conclusion

In light of the growing emergence of viral diseases in cetacean populations, methods other than visual assessment are needed to diagnose skin diseases and enable their use as potential health indicators. For this purpose, stranded cetaceans are outstanding resources for testing evidence-based approaches to identifying viruses from skin lesions. Future studies should combine macroscopic and histopathological studies of skin lesions with quantitative molecular analyses to further understand the epidemiology of viral skin diseases in cetacean wild populations.

## Data availability statement

The original contributions presented in the study are included in the article/Supplementary material, further inquiries can be directed to the corresponding author.

## Ethics statement

The requirement of ethical approval was waived by Environmental department of the Canary Islands Government and the Spanish Ministry of Environment for the studies involving animals because no experiments were performed on live animals. The studies were conducted in accordance with the local legislation and institutional requirements.

## Author contributions

ES, AF, CF, and MAn: conceptualization and review and editing. SS-G, ES, AF, MAr, MAB, CF, IF-J, and AC-R: methodology and formal analysis. SS-G, ES, and CF: writing – original draft preparation. ES, AF, MAr, CF, and MAB: supervision. AF, ES, and MAr: funding acquisition. All authors contributed to the article and approved the submitted version.

## Funding

This research was partially supported by a National Project (ref. PID2021-127687NB-I00); by the Canary Islands Government, which has founded and provided support to the stranding network; Agencia Canaria de Investigación, Innovación y Sociedad de la Información (ref. CEI2020-05); and by Fundación Loro Parque.

## Conflict of interest

The authors declare that the research was conducted in the absence of any commercial or financial relationships that could be construed as a potential conflict of interest.

## Publisher’s note

All claims expressed in this article are solely those of the authors and do not necessarily represent those of their affiliated organizations, or those of the publisher, the editors and the reviewers. Any product that may be evaluated in this article, or claim that may be made by its manufacturer, is not guaranteed or endorsed by the publisher.

## Footnotes

^1^ www.ncbi.nlm.nih.gov/blast/Blast.cgi
