# Supplementary material for: Viral skin diseases in odontocete cetaceans: gross, histopathological, and molecular characterization of selected pathogens
Source: Front Vet Sci. 2023 Sep 8;10:1188105. doi: 10.3389/fvets.2023.1188105 (PMC10514499; doi:10.3389/fvets.2023.1188105)
Supplement: Supplementary file 1 [file Table_1.pdf]

**Supplementary Table 1** Biological and stranding conditions of the 31 cetaceans included in the present study.

| CASE N. | ID CODE | SPECIES | AGE | SEX | SD | SL | SS | DC | CM |
|---|---|---|---|---|---|---|---|---|---|
| 1 | CET 566 | *S. coeruleoalba* | A | F | 26/03/2011 | Tenerife | D | 2 | F |
| 2 | CET 601 | *S. frontalis* | J | M | 05/02/2012 | Tenerife | D | 2 | RT |
| 3 | CET 642 | *S. frontalis* | A | F | 01/02/2013 | Lanzarote | A | 1 | RT |
| 4 | CET 663 | *D. delphis* | A | F | 06/05/2013 | Tenerife | A | 2 | RT |
| 5 | CET 705 | *S. coeruleoalba* | J | F | 23/03/2014 | Gran Canaria | A | 1 | R |
| 6 | CET 748 | *S. coeruleoalba* | J | M | 06/03/2015 | Lanzarote | A | 2 | RT |
| 7 | CET 751 | *G. griseus* | A | F | 16/03/2015 | Tenerife | D | 4 | F |
| 8 | CET 947 | *D. delphis* | J | M | 02/01/2019 | Fuerteventura | D | 4 | F |
| 9 | CET 951 | *S. coeruleoalba* | C | M | 23/01/2019 | La Palma | D | 2 | F |
| 10 | CET 959 | *S. coeruleoalba* | A | M | 19/02/2019 | Fuerteventura | D | 4 | F |
| 11 | CET 969 | *G. macrorhynchus* | C | M | 24/03/2019 | Tenerife | A | 1 | RT |
| 12 | CET 983 | *S. coeruleoalba* | J | M | 20/04/2019 | Gran Canaria | D | 2 | F |
| 13 | CET 984 | *G. griseus* | C | M | 26/04/2019 | Gran Canaria | A | 1 | RT |
| 14 | CET 985 | *S. coeruleoalba* | A | M | 27/04/2019 | Tenerife | D | 2 | RT |
| 15 | CET 991 | *S. coeruleoalba* | A | F | 09/05/2019 | Fuerteventura | D | 2 | R |
| 16 | CET 995 | *G. macrorhynchus* | J | M | 20/05/2019 | Gran Canaria | D | 2 | R |
| 17 | CET 1020 | *T. truncatus* | J | F | 09/08/2019 | Tenerife | D | 2 | RT |
| 18 | CET 1035 | *S. coeruleoalba* | J | F | 04/10/2019 | Fuerteventura | A | 2 | F |
| 19 | CET 1044 | *S. frontalis* | C | F | 05/12/2019 | Tenerife | D | 4 | RT |
| 20 | CET 1045 | *D. delphis* | A | F | 05/12/2019 | Fuerteventura | A | 2 | F |
| 21 | CET 1056 | *S. frontalis* | C | M | 24/01/2020 | Tenerife | D | 2 | RT |
| 22 | CET 1058 | *S. frontalis* | C | M | 27/01/2020 | Gran Canaria | D | 2 | F |
| 23 | CET 1067 | *S. frontalis* | C | F | 12/03/2020 | Tenerife | D | 2 | F |
| 24 | CET 1069 | *S. coeruleoalba* | A | M | 13/03/2020 | Gran Canaria | D | 2 | F |
| 25 | CET 1103 | *T. truncatus* | C | M | 13/06/2020 | Gran Canaria | A | 2 | RT |
| 26 | CET 1138 | *S. frontalis* | C | M | 18/12/2020 | Gran Canaria | D | 2 | F |
| 27 | CET 1151 | *T. truncatus* | C | M | 21/02/2021 | Tenerife | D | 2 | RT |
| 28 | CET 1152 | *S. frontalis* | C | M | 26/02/2021 | Gran Canaria | A | 1 | F |
| 29 | CET 1153 | *D. delphis* | A | M | 02/03/2021 | Gran Canaria | D | 2 | F |
| 30 | CET 1173 | *S. frontalis* | J | F | 17/04/2021 | Tenerife | D | 2 | RT |
| 31 | CET 1181 | *G. macrorhynchus* | C | F | 05/12/2021 | Tenerife | D | 2 | RT |

*AGE (A = adult, J = juvenile, C = calf, N = neonate); SEX (F = female, M = male); SD (stranding date); SL (stranding location); SS (stranding stage: A = alive; D = dead); DC (decomposition code); CM (conservation method); DC ( 1 = extremely fresh carcass, 2 = fresh carcass, 3 = moderate decomposition, 4 = advanced decomposition, and 5 = mummified or skeletal remains); CM (F = frozen, RT = room temperature)

**Supplementary Table 2** Gross classification and histopathology of the 55 skin lesions with their respective molecular results from the 31 animals of the present study.

| CASE N. | ID CODE | MACROSCOPIC CLASSIFICATION | | HISTOPATHOLOGICAL FINDINGS | MOLECULAR RESULTS | ASSOCIATED RAKE MARKS/WOUNDS |
|---|---|---|---|---|---|---|
| **1** | CET 566 | A1 | 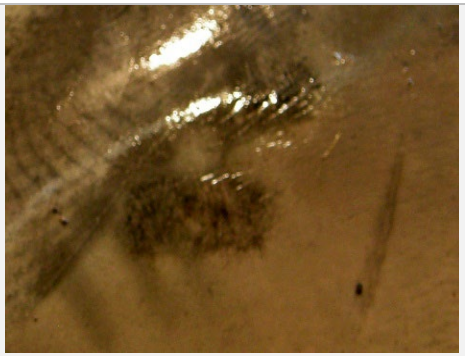<br>WHITE - FRINGED | NE | Negative | Yes |
| **2** | CET 601 | A1* | 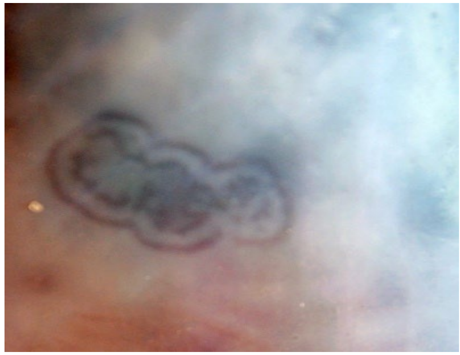<br>TATTOO-LIKE COALESCED | Minimal hyperkeratosis, acanthosis, and ballooning degeneration.<br><br>Mild intracellular edema. | CePV-1+ (ON600451) | No |

| | | | | | | |
|---|---|---|---|---|---|---|
| **3** | CET 642 | A1 | 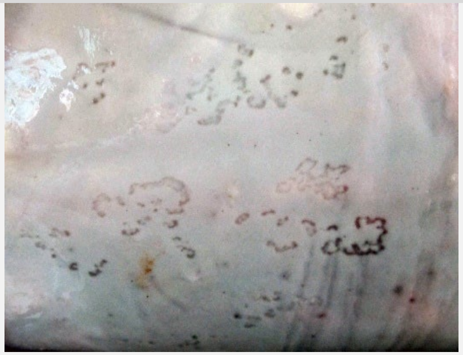 TATTOO-LIKE SERPIGINOUS | Minimal necrosis.<br><br>Mild acanthosis, intracellular edema, and inflammatory cell infiltration.<br><br>Moderate hyperkeratosis, ballooning degeneration and congestion.<br><br>Presence of ICIBs. | CePV-1+ (ON600452);<br>HV+ (OM456331) | No |
| **4** | CET 663 | A1 | 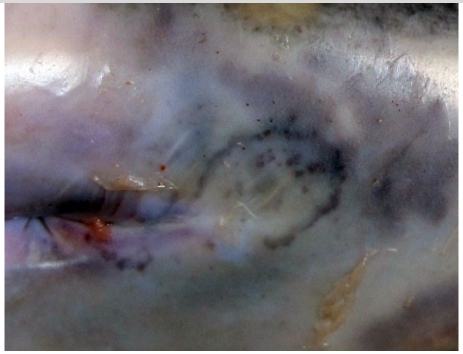 TATTOO-LIKE OVAL SHAPE | NE | CePV-1+ (ON600453) | No |
| **5** | CET 705 | A1 | 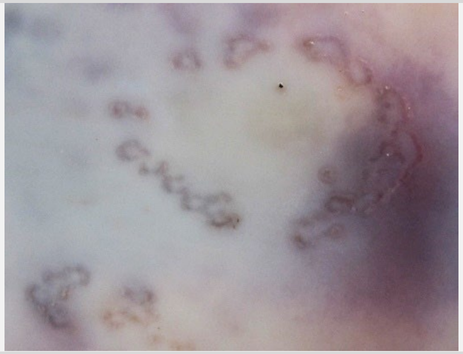 | Minimal ballooning degeneration and intracellular edema.<br><br>Mild hyperkeratosis and acanthosis.<br><br>Moderate congestion.<br><br>Presence of ICIBs. | CePV-1+ (ON600454) | No |

| | | | | | | |
|---|---|---|---|---|---|---|
| | | | TATTOO-LIKE SERPIGINOUS | | | |
| **6** | CET 748 | A1 | 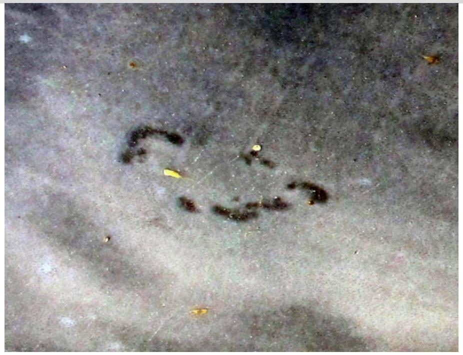<br>TATTOO-LIKE SERPIGINOUS | NE | CePV-1+ (ON600455) | Yes |
| **7** | CET 751 | A1 | 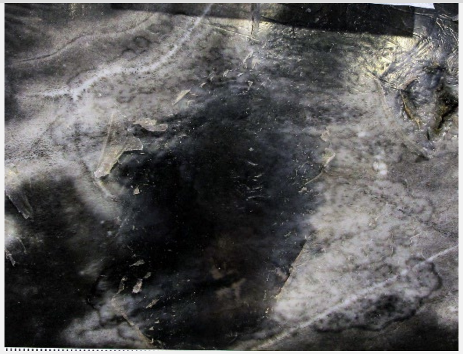<br>TATTOO-LIKE OVAL SHAPE | Minimal spongiosis, hyperpigmentation, fused rete ridges, inflammatory cell infiltration and dyskeratosis/apoptosis.<br>Mild acanthosis.<br>Moderate hyperkeratosis, ballooning degeneration and intracellular edema. | CePV-1+ (ON600456) | Yes |

| | | | | | | |
|---|---|---|---|---|---|---|
| **8** | CET 947 | A3 | 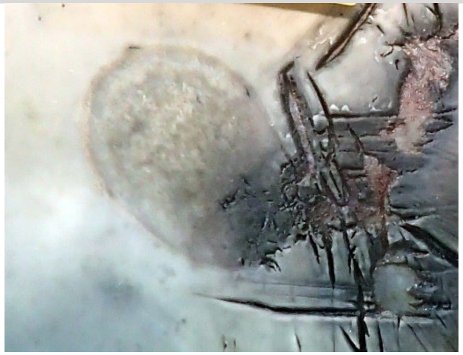 TATTOO-LIKE OVAL SHAPE | NE | CePV-1+ (ON600460); HV+ (OM456332) | Yes |
| **9** | CET 951 | A1 | 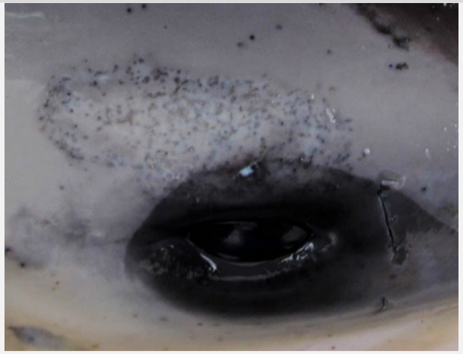 TATTOO-LIKE OVAL SHAPE | Minimal presence of pearl corns. Mild inflammatory cell infiltration and congestion. Moderate acanthosis, necrosis, and dyskeratosis/apoptosis. Presence of ICIBs and INIBs. Presence of satellitosis. | CePV-1+ (ON600461); HV+ (OM456333) | No |
| **10** | CET 959 | A1 | 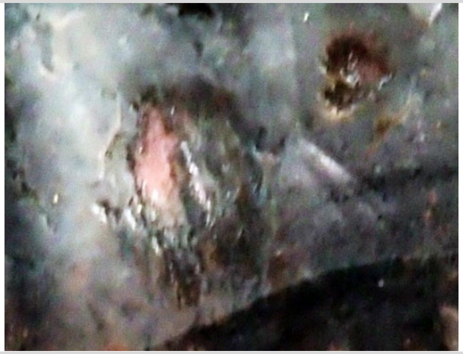 | Minimal inflammatory cell infiltration | Negative | Yes |

| | | | | | | |
|---|---|---|---|---|---|---|
| | | | ULCERATIVE | | | |
| **11** | CET 969 | A6 | 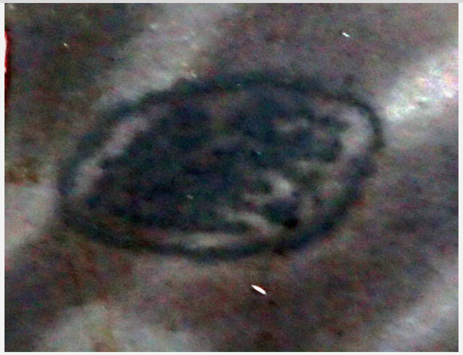 TATTOO-LIKE OVAL SHAPE | Minimal intracellular edema, inflammatory cell infiltration and congestion. Mild hyperkeratosis, acanthosis, and ballooning degeneration. Presence of ICIBs. | CePV-1+ (ON600462) | No |
| **12** | CET 983 | A3 | 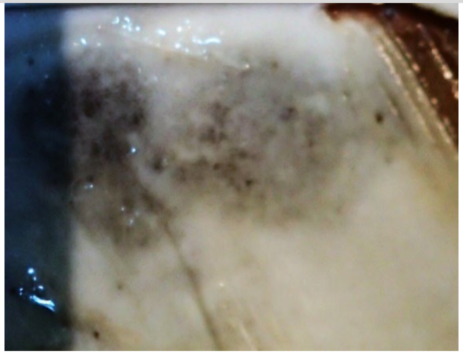 TATTOO-LIKE COALESCED | Minimal acanthosis, hyperpigmentation, inflammatory cell infiltration and presence of pearl corns. | CePV-1+ (ON600463) | Yes |

| | | | | | | |
|---|---|---|---|---|---|---|
| **13** | CET 984 | A4 | 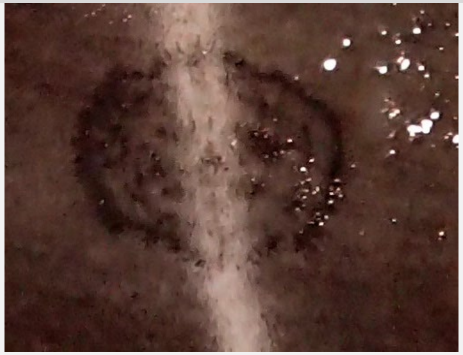 TATTOO-LIKE OVAL SHAPE | NA | CePV-1+ (ON600464);<br>HV+ (OM456334) | Yes |
| **14** | CET 985 | A1 | 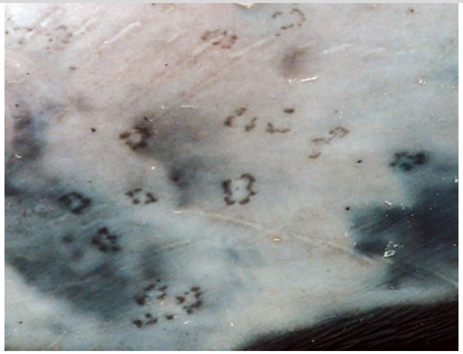 TATTOO-LIKE SERPIGINOUS | Minimal necrosis.<br>Mild hyperkeratosis, acanthosis, ballooning degeneration and intracellular edema.<br>Moderate congestion.<br>Presence of ICIBs. | CePV-1+ (ON600465);<br>HV+ (OM456335) | Yes |
| **15** | CET 991 | A3 | 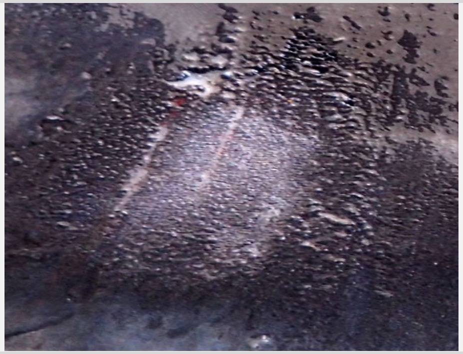 | Absence of associated histopathological changes | Negative | Yes |

| | | | | | | |
|---|---|---|---|---|---|---|
| | | | RING | | | |
| **16** | CET 995 | A1 | 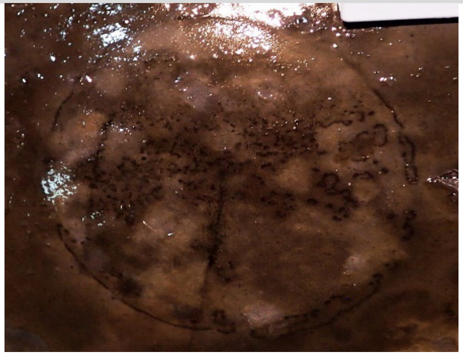<br>TATTOO-LIKE OVAL SHAPE | Minimal hyperkeratosis and ballooning degeneration.<br>Mild acanthosis, spongiosis, inflammatory cell infiltration and dyskeratosis/apoptosis.<br>Severe congestion. | CePV-1+ (ON600457) | Yes |
| **17** | CET 1020 | A1 | 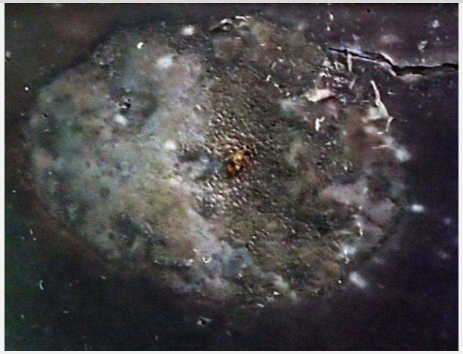<br>TATTOO-LIKE OVAL SHAPE | Minimal spongiosis, necrosis, fused rete ridges and inflammatory cell infiltration.<br>Mild hyperkeratosis and acanthosis, and dyskeratosis/apoptosis.<br>Moderate ballooning degeneration and intracellular edema.<br>Presence of ICIBs. | CePV-1+ (ON600466);<br>HV+ (OM456336) | No |

| | | | | | | |
|---|---|---|---|---|---|---|
| **18** | CET 1035 | A2 | 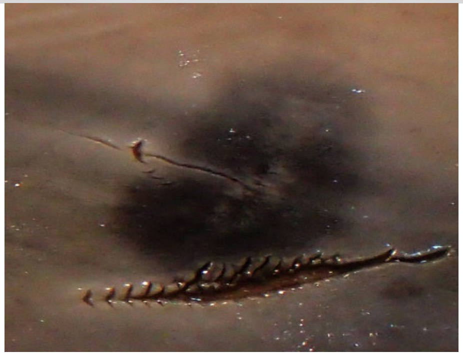<br>BLACK-FRINGED | Minimal hyperkeratosis and inflammatory cell infiltration.<br><br>Mild acanthosis.<br><br>Moderate fused rete ridges. | CeMV+ (ON314830) | Yes |
| **19** | CET 1044 | A1 | 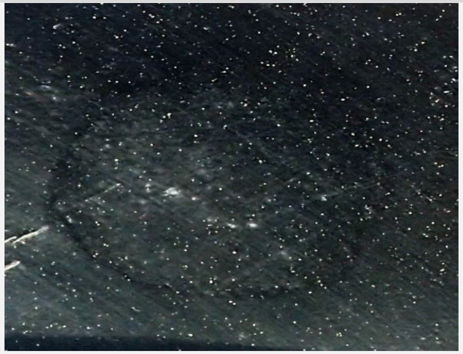<br>RING | NA | HV+ (OM456337) | Yes |
| **20** | CET 1045 | A4 | 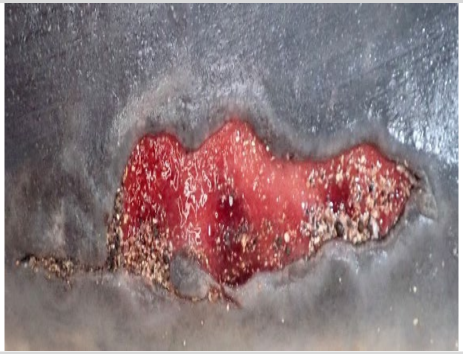 | NA | HV+ (OM456338) | Yes |

| | | | | | | |
|---|---|---|---|---|---|---|
| | | | ULCERATIVE | | | |
| **21** | CET 1056 | A1 | 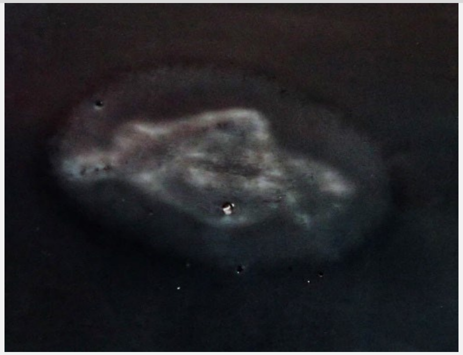 BLACK-FRINGED | Minimal fused rete ridges and inflammatory cell infiltration.<br><br>Mild hypopigmentation and congestion.<br><br>Moderate acanthosis. | HV+ (OM456339) | No |
| **22** | CET 1058 | A1 | 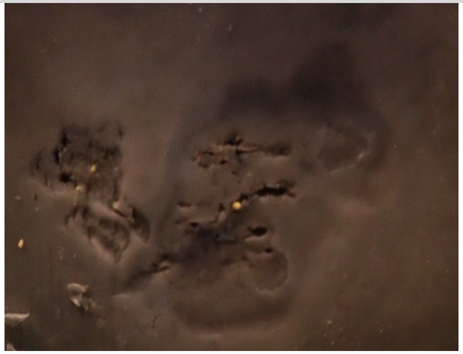 BLACK-FRINGED | Minimal ballooning degeneration, intracellular edema, and congestion.<br><br>Mild inflammatory cell infiltration.<br><br>Moderate necrosis. | CePV-1+ (ON600467);<br><br>HV+ (OM456340) | No |

| | | | | | | |
|---|---|---|---|---|---|---|
| **23** | CET 1067 | A3 | 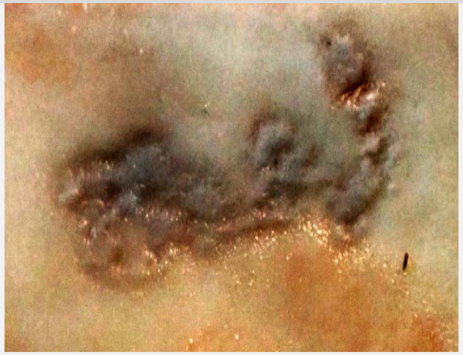<br>TORTUOUS | Mild acanthosis, hypopigmentation, and presence of pearl corns.<br><br>Moderate hyperkeratosis, necrosis, fused rete ridges and inflammatory cell infiltration.<br><br>Presence of ICIBs. | HV+ (ON314829) | No |
| **24** | CET 1069 | A1 | 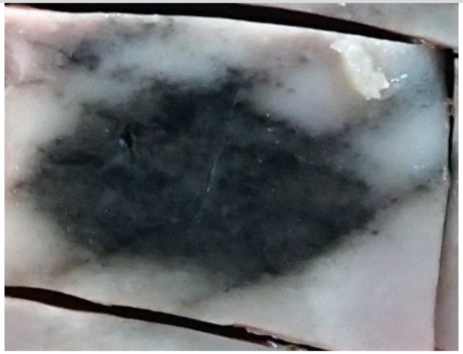<br>RING | NE | CePV-1+ (ON600468) | Yes |
| **25** | CET 1103 | A2* | 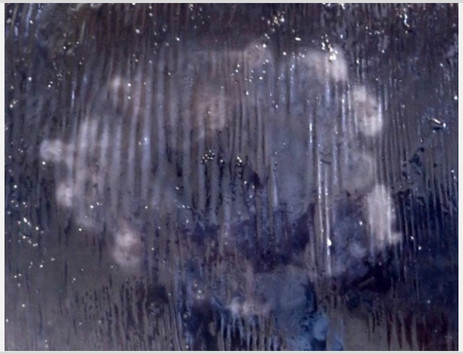 | Minimal necrosis, hyperpigmentation, and hypopigmentation.<br><br>Mild hyperkeratosis, intracellular edema, and congestion.<br><br>Moderate acanthosis and inflammatory cell infiltration.<br><br>Severe fused rete ridges. | HV+ (OM456341) | No |

| | | | | | | |
|---|---|---|---|---|---|---|
| | | | PALE | Presence of INIBs. | | |
| **26** | CET 1138 | A1 | 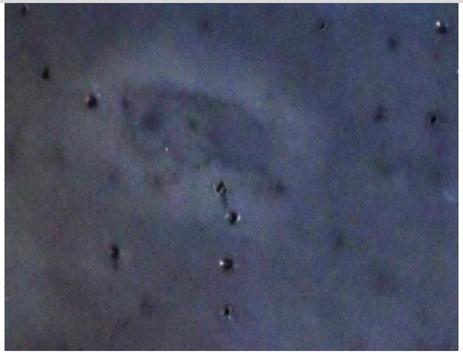 WHITE-FRINGED | NE | HV+ (OM456342) | Yes |
| | | A2 | 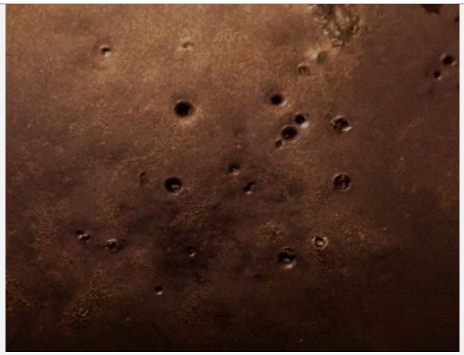 WHITE-FRINGED | NA | HV+ (OM456342) | Yes |

| | | | | | | |
|---|---|---|---|---|---|---|
| | | A3 | 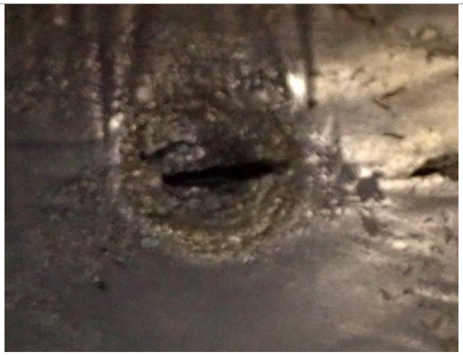<br>TARGET-LIKE | NE | HV+ (OM456342) | Yes |
| | | A4 | 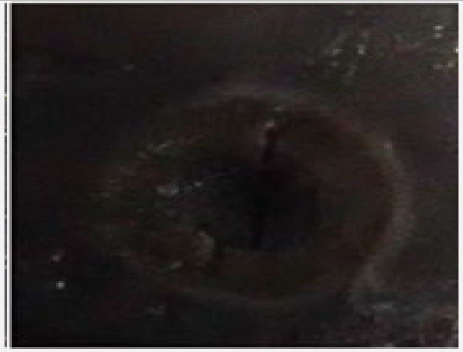<br>TARGET-LIKE | NE | Negative | Yes |
| | | A5 | 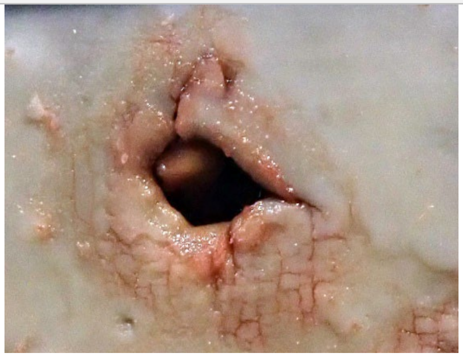<br>ULCERATIVE | NE | HV+ (OM456342) | No |

| | | | | | | |
|---|---|---|---|---|---|---|
| **27** | CET 1151 | A1* | Image rights not deserved.<br>*Image previously reported in: "Segura-Göthlin, S., A. Fernández, M. Arbelo, I. Felipe-Jiménez, A. Colom-Rivero, J. Almunia, and E. Sierra, 2021: The Validation of a Non-Invasive Skin Sampling Device for Detecting Cetacean Poxvirus. Anim. 2021, Vol. 11, Page 2814* ***11****, 2814, DOI: 10.3390/ANI11102814".*<br><br>TATTOO-LIKE SERPIGINOUS | Minimal intracellular edema and fused rete ridges.<br><br>Mild acanthosis, ballooning degeneration and congestion.<br><br>Moderate hyperkeratosis.<br><br>Presence of ICIBs. | CePV-1+ (ON600458);<br><br>HV+ (OM456343) | No |
| | | A3 | 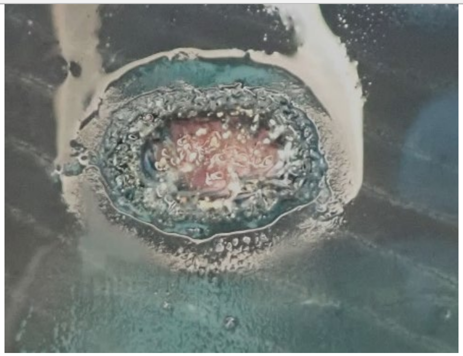<br>TARGET-LIKE | NA | HV+ (OM456344) | Yes |
| | | A4 | 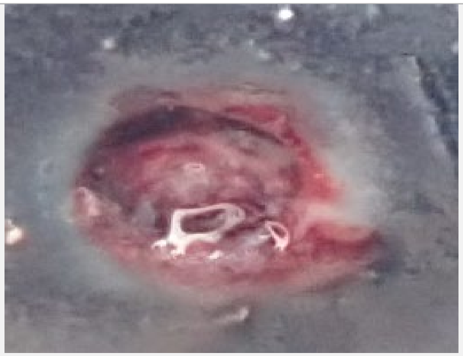<br>ULCERATIVE | NA | HV+ (OM456343) | No |
| | | A6* | Image rights not deserved.<br>*Image previously reported in: "Segura-Göthlin, S., A. Fernández, M. Arbelo, I. Felipe-Jiménez, A. Colom-Rivero, J. Almunia, and E. Sierra, 2021: The Validation of a Non-Invasive Skin Sampling Device for Detecting Cetacean* | Minimal intracellular edema and pearl corns. | CePV-1+ (ON600458);<br>HV+ (OM456344) | No |

| | | | | | | |
|---|---|---|---|---|---|---|
| | | | *Poxvirus. Anim. 2021, Vol. 11, Page 2814* ***11****, 2814, DOI: 10.3390/ANI11102814".*<br><br>RING | Mild hyperkeratosis, ballooning degeneration and acanthosis.<br><br>Presence of ICIBs. | | |
| **28** | CET 1152 | A1 | 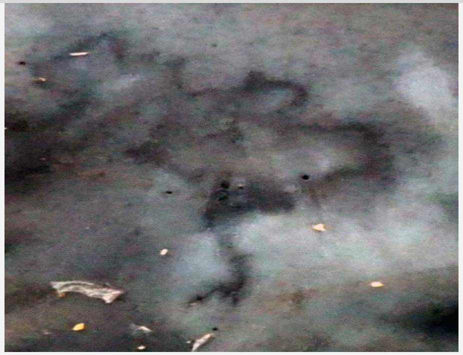<br>TORTUOUS | Minimal hyperkeratosis, acanthosis, necrosis, and inflammatory cell infiltration. | HV+ (OM456345) | No |
| | | A2 | 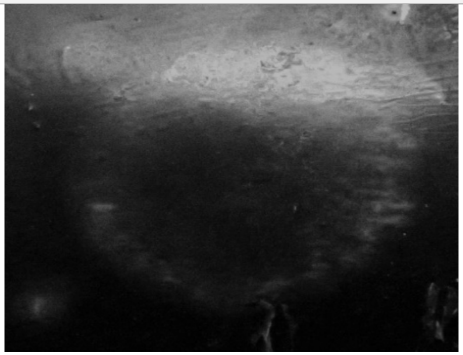<br>WHITE-FRINGED | Minimal hyperkeratosis, necrosis, fused rete ridges, inflammatory cell infiltration, congestion, and pearl corns.<br><br>Mild acanthosis. | HV+ (OM456345) | No |

| | | | | | | |
|---|---|---|---|---|---|---|
| | | A3 | 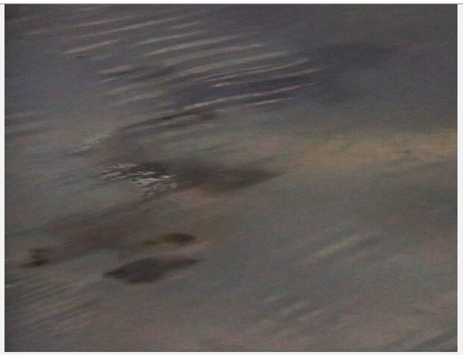 TORTUOUS | Mild hyperkeratosis and acanthosis.<br><br>Moderate necrosis and inflammatory cell infiltration. | Negative | No |
| | | A4 | 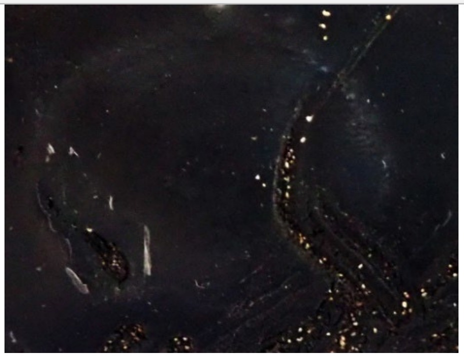 RING | Minimal acanthosis, intracellular edema, inflammatory cell infiltration and congestion. | HV+ (OM456345) | Yes |
| | | A5 | 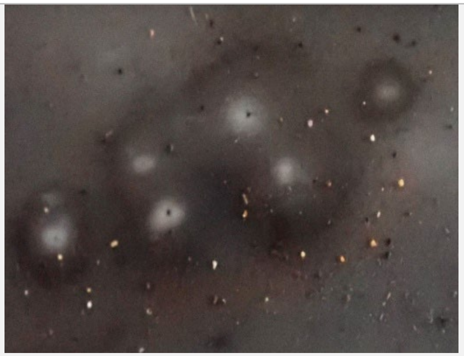 BLACK-FRINGED | Minimal intracellular edema and dyskeratosis/apoptosis.<br><br>Mild hyperkeratosis, acanthosis, and necrosis.<br><br>Moderate inflammatory cell infiltration. | HV+ (OM456345) | No |

| | | | | | | |
|---|---|---|---|---|---|---|
| | | A1 | 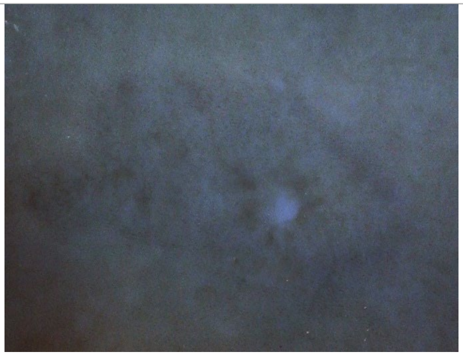 BLACK-FRINGED | Minimal inflammatory cell infiltration.<br><br>Moderate hyperkeratosis, acanthosis, and fused rete ridges. | Negative | No |
| **29** | CET 1153 | A2 | 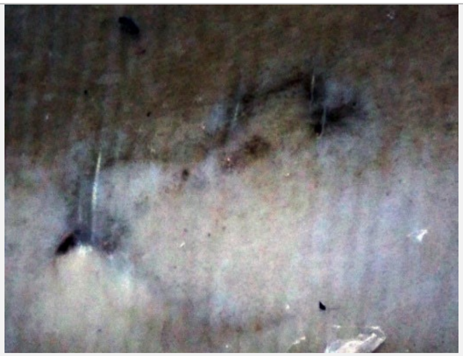 TORTUOUS | Minimal inflammatory cell infiltration and congestion.<br><br>Mild hyperkeratosis, acanthosis, and fused rete ridges. | Negative | No |
| | | A3 | 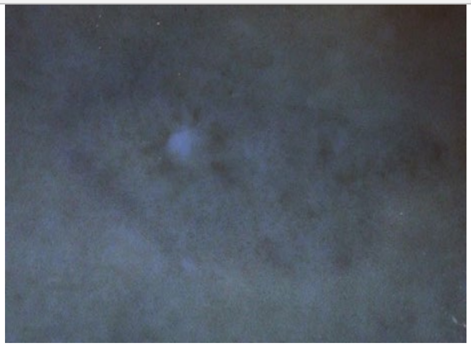 BLACK-FRINGED | Minimal acanthosis, inflammatory cell infiltration, congestion, and dyskeratosis/apoptosis.<br><br>Moderate fused rete ridges. | Negative | No |

| | | | | | | |
|---|---|---|---|---|---|---|
| **30** | CET 1173 | A1* | Image rights not deserved.<br>*Image previously reported in: "Segura-Göthlin, S., A. Fernández, M. Arbelo, I. Felipe-Jiménez, A. Colom-Rivero, J. Almunia, and E. Sierra, 2021: The Validation of a Non-Invasive Skin Sampling Device for Detecting Cetacean Poxvirus. Anim. 2021, Vol. 11, Page 2814* ***11****, 2814, DOI: 10.3390/ANI11102814".*<br><br>TATTOO-LIKE OVAL SHAPE | Minimal congestion.<br><br>Mild acanthosis, intracellular edema, hyperpigmentation, and inflammatory cell infiltration.<br><br>Moderate hyperkeratosis and ballooning degeneration.<br><br>Presence of ICIBs. | CePV-1+ (ON600459) | No |
| | | A2* | | Minimal congestion.<br><br>Mild acanthosis, intracellular edema, hyperpigmentation, and inflammatory cell infiltration.<br><br>Moderate hyperkeratosis and ballooning degeneration.<br><br>Presence of ICIBs. | CePV-1+ (ON600459) | No |
| | | A3* | | Minimal congestion.<br><br>Mild acanthosis, intracellular edema, hyperpigmentation, and inflammatory cell infiltration.<br><br>Moderate hyperkeratosis and ballooning degeneration.<br><br>Presence of ICIBs. | CePV-1+ (ON600459) | No |
| | | A4* | Image rights not deserved.<br>*Image previously reported in: "Segura-Göthlin, S., A. Fernández, M. Arbelo, I. Felipe-Jiménez, A. Colom-Rivero, J. Almunia, and E. Sierra, 2021: The Validation of a Non-Invasive Skin Sampling Device for Detecting Cetacean Poxvirus. Anim. 2021, Vol. 11, Page 2814* ***11****, 2814, DOI: 10.3390/ANI11102814".*<br><br>BLACK-FRINGED | Minimal acanthosis, ballooning degeneration, intracellular edema, congestion, and dyskeratosis/apoptosis.<br><br>Mild hyperkeratosis, spongiosis, and inflammatory cell infiltration. | CePV-1+ (ON600459) | No |

| | | | | | | |
|---|---|---|---|---|---|---|
| | | A5* | | Minimal ballooning degeneration and dyskeratosis/apoptosis.<br><br>Mild acanthosis, hyperpigmentation, and congestion.<br><br>Moderate hyperkeratosis and inflammatory cell infiltration. | CePV-1+ (ON600459) | No |
| | | A6* | Image rights not deserved.<br>*Image previously reported in: "Segura-Göthlin, S., A. Fernández, M. Arbelo, I. Felipe-Jiménez, A. Colom-Rivero, J. Almunia, and E. Sierra, 2021: The Validation of a Non-Invasive Skin Sampling Device for Detecting Cetacean Poxvirus. Anim. 2021, Vol. 11, Page 2814* ***11****, 2814, DOI: 10.3390/ANI11102814".*<br><br>WHITE-FRINGED | Minimal hyperkeratosis, acanthosis, hyperpigmentation, and inflammatory cell infiltration. | CePV-1+ (ON600459) | No |
| | | A7* | | Minimal acanthosis, inflammatory cell infiltration, and congestion. | CePV-1+ (ON600459) | No |
| | | A8* | Image rights not deserved.<br>*Image previously reported in: "Segura-Göthlin, S., A. Fernández, M. Arbelo, I. Felipe-Jiménez, A. Colom-Rivero, J. Almunia, and E. Sierra, 2021: The Validation of a Non-Invasive Skin Sampling Device for Detecting Cetacean Poxvirus. Anim. 2021, Vol. 11, Page 2814 11, 2814, DOI: 10.3390/ANI11102814".*<br><br>RING | NA | CePV-1+ (ON600459) | No |
| | | A9* | Image rights not deserved.<br>*Image previously reported in: "Segura-Göthlin, S., A. Fernández, M. Arbelo, I. Felipe-Jiménez, A. Colom-Rivero, J. Almunia, and E. Sierra, 2021: The Validation of a Non-Invasive Skin Sampling Device for Detecting Cetacean Poxvirus. Anim. 2021, Vol. 11, Page 2814 11, 2814, DOI: 10.3390/ANI11102814".*<br><br>WHITE-FRINGED | Minimal acanthosis, fused rete ridges, and inflammatory cell infiltration.<br><br>Mild congestion. | CePV-1+ (ON600459) | No |
| | | A10* | | NA | CePV-1+ (ON600459) | No |

| | | | | | | |
|---|---|---|---|---|---|---|
| **31** | CET 1181 | A1 | 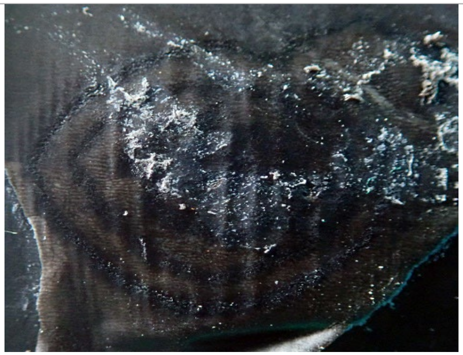<br>TATTOO-LIKE OVAL SHAPE | Minimal acanthosis and dyskeratosis/apoptosis.<br><br>Mild hyperkeratosis and ballooning degeneration.<br><br>Presence of ICIBs. | CePV-1+ (ON600469) | No |
| | | A2 | 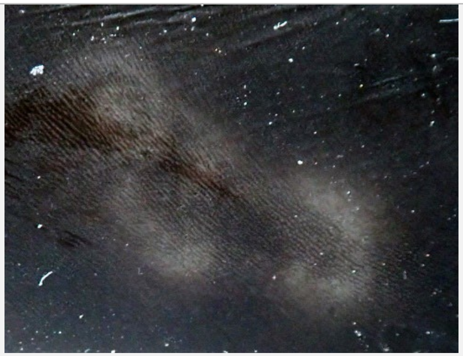<br>TATTOO-LIKE COALESCED | Minimal ballooning degeneration. | Negative | No |
| | | A3 | 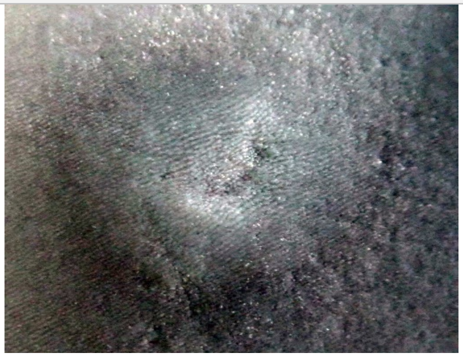<br>BLACK-FRINGED | Minimal hyperkeratosis, acanthosis, and inflammatory cell infiltration. | CePV-1+ (ON600469) | No |

*CePV, cetacean poxvirus; HV, herpesvirus; CeMV, cetacean morbillivirus; A1 – A10 = skin lesion samples 1 to 10; NA, not applicable; NE, not evaluated; +, positive; -, negative. Asterisks indicate positive cases that have been previously published.

**Supplementary Table 3.** Percentage of identity of the sequences from the present study with the closest available ones in GenBank.

| CASE N. | ID CODE | SKIN LESION | SEQUENCES | | | | | | | | | | | |
|---|---|---|---|---|---|---|---|---|---|---|---|---|---|---|
| | | | CePV | | | | | | HV | | | | | |
| | | | Accession Number | Bp | Organism | Percentage Identity | Isolation Source | References | Accession Number | Bp | Organism | Percentage Identity | Isolation Source | References |
| **1** | CET 566 | A1 | - | - | - | - | - | - | - | - | - | - | - | - |
| **2** | CET 601 | A1 | ON600451 | 497 | CePV-1 | 93.75% (OQ102395) | Skin | NA | - | - | - | - | - | - |
| **3** | **CET 642** | **A1** | **ON600452** | **497** | **CePV-1** | **93.75% (OQ102395)** | **Skin** | **NA** | **OM456331** | **193** | **Alphaherpesvirus** | **98.45% (MG437205)** | **NA** | **NA** |
| **4** | CET 663 | A1 | ON600453 | 497 | CePV-1 | 100% (KC409046) | Skin | (1) | - | - | - | - | - | - |
| **5** | CET 705 | A1 | ON600454 | 497 | CePV-1 | 99.80% (KC409049) | Skin | (1) | - | - | - | - | - | - |
| **6** | CET 748 | A1 | ON600455 | 497 | CePV-1 | 99.80% (KC409049) | Skin | (1) | - | - | - | - | - | - |
| **7** | CET 751 | A1 | ON600456 | 497 | CePV-1 | 95.97% (KC409049) | Skin | (1) | - | - | - | - | - | - |
| **8** | **CET 947** | **A3** | **ON600460** | **98** | **CePV-1** | **96.91% (MH005249)** | **Skin** | (2) | **OM456332** | **193** | **Alphaherpesvirus** | **100% (MG437205)** | **NA** | **NA** |
| **9** | **CET 951** | **A1** | **ON600461** | **85** | **CePV-1** | **90% (AY463006)** | **Skin** | (3) | **OM456333** | **193** | **Alphaherpesvirus** | **97.41% (MG437205)** | **NA** | **NA** |
| **10** | CET 959 | A1 | - | - | - | - | - | - | - | - | - | - | - | - |
| **11** | CET 969 | A6 | ON600462 | 98 | CePV-1 | 96.91% (MH005249) | Skin | (2) | - | - | - | - | - | - |
| **12** | CET 983 | A3 | ON600463 | 98 | CePV-1 | 96.91 (MH005249) | Skin | (2) | - | - | - | - | - | - |
| **13** | **CET 984** | **A4** | **ON600464** | **98** | **CePV-1** | **93.81% (MH005249)** | **Skin** | **(2)** | **OM456334** | **181** | **Alphaherpesvirus** | **100% (KP995683)** | **Brain** | **(4)** |
| **14** | **CET 985** | **A1** | **ON600465** | **98** | **CePV-1** | **96.91% ( MH005249)** | **Skin** | **(2)** | **OM456335** | **169** | **Gammaherpesvirus** | **100% (KM248274)** | **Penis** | **(5)** |
| **15** | CET 991 | A3 | - | - | - | - | - | - | - | - | - | - | - | - |
| **16** | CET 995 | A1 | ON600457 | 497 | CePV-1 | 94.96% (KC409049) | Skin | (1) | - | - | - | - | -- | - |
| **17** | **CET 1020** | **A1** | **ON600466** | **98** | **CePV-1** | **96.94% (MH005249)** | Skin | (2) | **OM456336** | **194** | **Alphaherpesvirus** | **96.91% (MG437205)** | **NA** | **NA** |
| **18** | CET 1035 | A2 | - | - | - | - | - | - | - | - | - | - | | - |
| **19** | CET 1044 | A1 | - | - | - | - | - | - | OM456337 | 190 | Alphaherpesvirus | 100% (MN179657) | Brain | NA |

| | | | | | | | | | | | | | | |
|---|---|---|---|---|---|---|---|---|---|---|---|---|---|---|
| **20** | CET 1045 | A4 | - | - | - | - | - | - | OM456338 | 181 | Alphaherpesvirus | 100% ( MN179655) | Brain | NA |
| **21** | CET 1056 | A1 | - | - | - | - | - | - | OM456339 | 190 | Alphaherpesvirus | 100% (MN179657) | Brain | NA |
| **22** | **CET 1058** | **A1** | **ON600467** | **82** | **CePV-1** | **92.68% (AY952950)** | **Skin** | (3) | **OM456340** | **193** | **Alphaherpesvirus** | **98.45% (MG437205)** | **NA** | **NA** |
| **23** | CET 1067 | A3 | - | - | - | - | - | - | ON314829 | 169 | Gammaherpesvirus | 98.42% (KM248274) | Penis | (5) |
| **24** | CET 1069 | A1 | ON600468 | 75 | CePV-1 | 92.11% (AY952950) | Skin | (3) | - | - | - | - | - | - |
| **25** | CET 1103 | A2 | - | - | - | - | - | - | OM456341 | 190 | Alphaherpesvirus | 100% (MG437217) | NA | NA |
| | | A1 | | | | | | | | | | | | |
| | | A2 | | | | | | | OM456342 | 190 | Alphaherpesvirus | 99.47% (MN179657) | Brain | NA |
| **26** | CET 1138 | A3 | - | - | - | - | - | - | | | | | | |
| | | A4 | | | | | | | - | - | - | - | - | - |
| | | A5 | | | | | | | OM456342 | 190 | Alphaherpesvirus | 99.47% (MN179657) | Brain | NA |
| | | **A1** | **ON600458** | **497** | **CePV-1** | **100% (OQ102395)** | **Skin** | **-.** | **OM456343** | **190** | **Alphaherpesvirus** | **99.47% (AY949832)** | **Skin** | **(6)** |
| **27** | CET 1151 | A3 | - | - | - | - | - | - | OM456344 | 190 | Alphaherpesvirus | 100% (AY608707) | Skin | (6) |
| | | A4 | | | | | | | OM456343 | 190 | Alphaherpesvirus | 99.47% (AY949832) | Skin | (6) |
| | | **A6** | **ON600458** | **497** | **CePV-1** | **100% (OQ102395)** | **Skin** | **-** | **OM456344** | **190** | **Alphaherpesvirus** | **100% (AY608707)** | **Skin** | **(6)** |
| | | A1 | | | | | | | OM456345 | 193 | Alphaherpesvirus | 98.45% (MG437205) | NA | NA |
| | | A2 | | | | | | | OM456345 | | | | | |
| **28** | CET 1152 | A3 | - | - | - | - | - | - | - | - | - | - | - | - |
| | | A4 | | | | | | | OM456345 | 193 | Alphaherpesvirus | 98.45% (MG437205) | NA | NA |
| | | A5 | | | | | | | OM456345 | | | | | |
| | | A1 | | | | | | | | | | | | |
| **29** | CET 1153 | A2 | - | - | - | - | - | - | - | - | - | - | - | - |
| | | A3 | | | | | | | | | | | | |
| **30** | CET 1173 | A1 A2 | ON600459 | 497 | CePV-1 | 98.99% (MF458199) | Skin | - | - | - | - | - | - | - |

| | | | | | | | | | | | | | | |
|---|---|---|---|---|---|---|---|---|---|---|---|---|---|---|
| | | A3 | | | | | | | | | | | | |
| | | A4 | | | | | | | | | | | | |
| | | A5 | | | | | | | | | | | | |
| | | A6 | | | | | | | | | | | | |
| | | A7 | | | | | | | | | | | | |
| | | A8 | | | | | | | | | | | | |
| | | A9 | | | | | | | | | | | | |
| | | A10 | | | | | | | | | | | | |
| **31** | CET 1181 | A1 | ON600469 | 77 | CePV-1 | 93.51% (AY952950) | Skin | (3) | - | - | - | - | - | - |
| | | A2 | - | - | - | - | - | - | | | | | | |
| | | A3 | ON600469 | 77 | CePV-1 | 93.51% (AY952950) | Skin | (3) | | | | | | |

*Bp, base pairs; CePV-1, cetacean poxvirus 1; HV, herpesvirus; NA, not available; -, absent.

**Supplementary Table 4** Most prevalent histologic findings in skin lesions of the present study.

| CASE N. | ID CODE | LESION | MC | MOST PREVALENT HISTOLOGICAL FINDINGS | | | | | | | | | | | | | | | |
|---|---|---|---|---|---|---|---|---|---|---|---|---|---|---|---|---|---|---|---|
| | | | | Hk | At | BD | Sp | IE | Ne | St | Hp | Ho | FRR | ICIBs | INIBs | ICI | Cg | Dk/Ap | PC |
| **2** | CET 601 | A1 | TL-C | + | + | + | - | ++ | - | no | - | - | - | no | no | - | - | - | - |
| **3** | CET 642 | A1 | TL-S | +++ | ++ | +++ | - | ++ | + | no | - | - | - | yes | no | ++ | +++ | - | - |
| **5** | CET 705 | A1 | TL-S | ++ | ++ | + | - | + | -- | - | - | - | - | no | no | - | +++ | - | - |
| **7** | CET 751 | A1 | TL-O | +++ | ++ | +++ | + | +++ | - | no | + | - | + | yes | no | + | - | + | - |
| **9** | CET 951 | A1 | TL-O | - | +++ | - | - | - | +++ | yes | - | - | - | yes | yes | ++ | ++ | +++ | + |
| **10** | CET 959 | A1 | U | - | - | NE | NE | NE | - | no | - | - | - | no | no | + | - | - | - |
| **11** | CET 969 | A6 | TL-O | ++ | ++ | ++ | - | + | - | no | - | - | - | yes | no | + | + | - | - |
| **12** | CET 983 | A3 | TL-C | NE | + | NE | NE | NE | - | no | + | - | - | no | no | + | - | - | + |
| **14** | CET 985 | A1 | TL-S | ++ | ++ | ++ | - | ++ | + | no | - | - | - | yes | no | - | +++ | - | - |
| **15** | CET 991 | A3 | R | NE | - | - | - | - | - | no | - | - | - | no | no | - | - | - | - |
| **16** | CET 995 | A1 | TL-O | + | ++ | + | ++ | NE | - | no | - | - | - | no | no | ++ | ++++ | ++ | - |
| **17** | CET 1020 | A1 | TL-O | ++ | ++ | +++ | + | +++ | + | no | - | - | + | yes | no | + | - | ++ | - |
| **18** | CET 1035 | A2 | BF | + | ++ | NE | NE | NE | - | no | - | - | +++ | no | no | + | - | - | - |
| **21** | CET 1056 | A1 | BF | - | +++ | - | - | - | - | no | - | ++ | + | no | no | + | ++ | - | - |
| **22** | CET 1058 | A1 | BF | NE | NE | + | - | + | +++ | no | - | - | - | no | no | ++ | + | - | - |
| **23** | CET 1067 | A3 | Ts | +++ | ++ | NE | NE | NE | +++ | yes | - | ++ | +++ | no | no | +++ | - | - | ++ |
| **25** | CET 1103 | A2 | P | ++ | +++ | - | - | ++ | + | no | + | + | ++++ | no | yes | +++ | ++ | - | - |
| **27** | CET 1151 | A1 | TL-S | +++ | ++ | ++ | - | + | - | - | - | - | + | yes | no | + | ++ | - | - |
| | | A6 | R | ++ | ++ | ++ | - | + | - | no | - | - | - | yes | no | - | - | - | + |
| **28** | CET 1152 | A1 | Ts | + | + | - | - | - | + | no | - | - | - | no | no | + | - | - | - |
| | | A2 | WF | + | ++ | - | - | - | + | no | - | - | + | no | no | + | + | - | + |
| | | A3 | Ts | ++ | ++ | - | - | - | +++ | no | - | - | - | no | no | +++ | - | - | - |
| | | A4 | R | - | + | - | - | + | - | no | - | - | - | no | no | + | + | - | - |

| | | | | | | | | | | | | | | | | | | | |
|---|---|---|---|---|---|---|---|---|---|---|---|---|---|---|---|---|---|---|---|
| | | A5 | BF | ++ | ++ | - | - | + | ++ | no | - | - | - | no | no | +++ | - | + | - |
| **29** | CET 1153 | A1 | BF | +++ | +++ | - | - | - | - | no | - | - | +++ | no | no | + | - | - | - |
| | | A2 | Ts | ++ | ++ | - | - | - | - | no | - | - | ++ | no | no | + | + | - | - |
| | | A3 | BF | - | ++ | - | - | - | - | no | - | - | +++ | no | no | + | + | + | - |
| **30** | CET 1173 | A1 | TL-O | +++ | ++ | +++ | - | ++ | - | no | ++ | - | - | yes | no | ++ | + | - | - |
| | | A2 | TL-O | +++ | ++ | +++ | - | ++ | - | no | ++ | - | - | yes | no | ++ | + | - | - |
| | | A3 | TL-O | +++ | +++ | ++++ | ++++ | +++ | - | no | + | - | - | yes | no | - | ++ | - | - |
| | | A4 | BF | ++ | + | + | + | + | - | no | - | - | - | no | no | ++ | + | + | - |
| | | A5 | BF | +++ | ++ | + | + | - | - | no | ++ | - | - | no | no | +++ | ++ | + | - |
| | | A6 | WF | + | + | - | - | - | - | no | + | - | - | no | no | + | - | - | - |
| | | A7 | WF | NE | + | - | - | - | - | no | - | - | - | no | no | + | + | - | - |
| | | A9 | WF | NE | + | - | - | - | - | no | - | - | + | no | no | + | ++ | - | - |
| **31** | CET 1181 | A1 | TL-O | ++ | + | ++ | - | - | - | no | - | - | - | yes | no | - | - | + | - |
| | | A2 | TL-C | - | - | + | - | - | - | no | - | - | - | no | no | + | - | - | - |
| | | A3 | BF | + | + | - | - | - | - | no | - | - | - | no | no | + | - | - | - |

*Hk, hyperkeratosis; At, acanthosis; Hp, hyperplasia; BD, ballooning degeneration; Sg, spongiosis; IE, intracytoplasmic oedema; MC, macroscopic classification; Nc, necrosis; St, satellistosis; Hp; hyperpigmentation; Ho, hypopigmentation; FRR, fused rete ridges; ICIBs, inclusion bodies; INIBs, intranuclear inclusion bodies; ICI, inflammatory cell infiltration; Cg, congestion; Dk/Ap, dyskeratosis/apoptosis; PC, pearl corns; A1 – A9 = skin lesion samples 1 to 9; BF (black-fringed); R (ring); T (target-like); Ts (tortuous); TL-C (tattoo-like, coalesced); TL-S (tattoo-like, serpiginous); TL-O (tattoo-like, oval-shaped); P (pale); U (ulcerative); WF (white-fringed); NE, not evaluable; -, absent; +, minimal; ++, mild; +++, moderate; ++++, severe.
