# Supplementary figures and images for: Viral skin diseases in odontocete cetaceans: gross, histopathological, and molecular characterization of selected pathogens
Source: Front Vet Sci. 2023 Sep 8;10:1188105. doi: 10.3389/fvets.2023.1188105 (PMC10514499; doi:10.3389/fvets.2023.1188105)

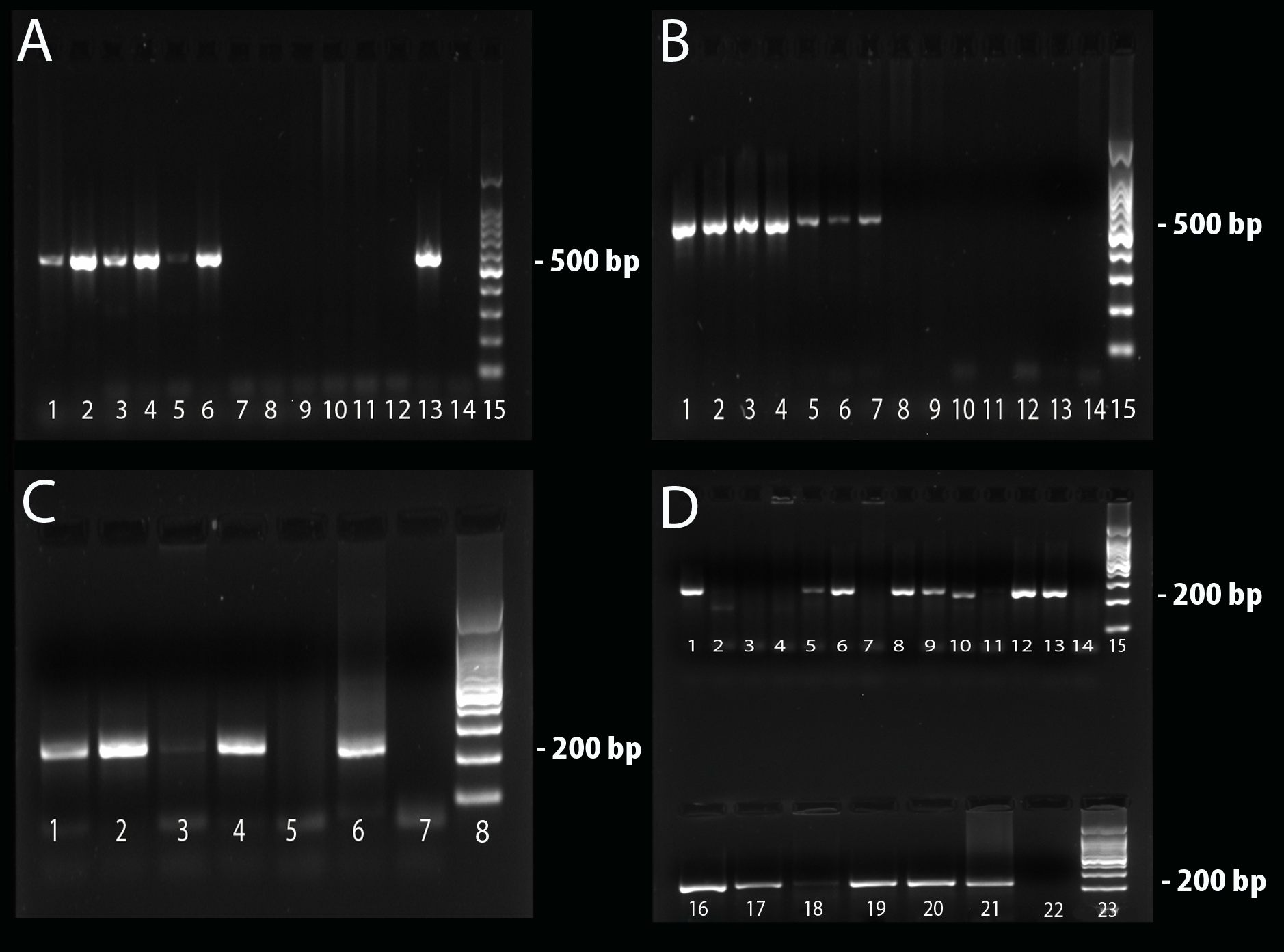

Supplement: Supplementary file 2 [file Image_1.TIF]

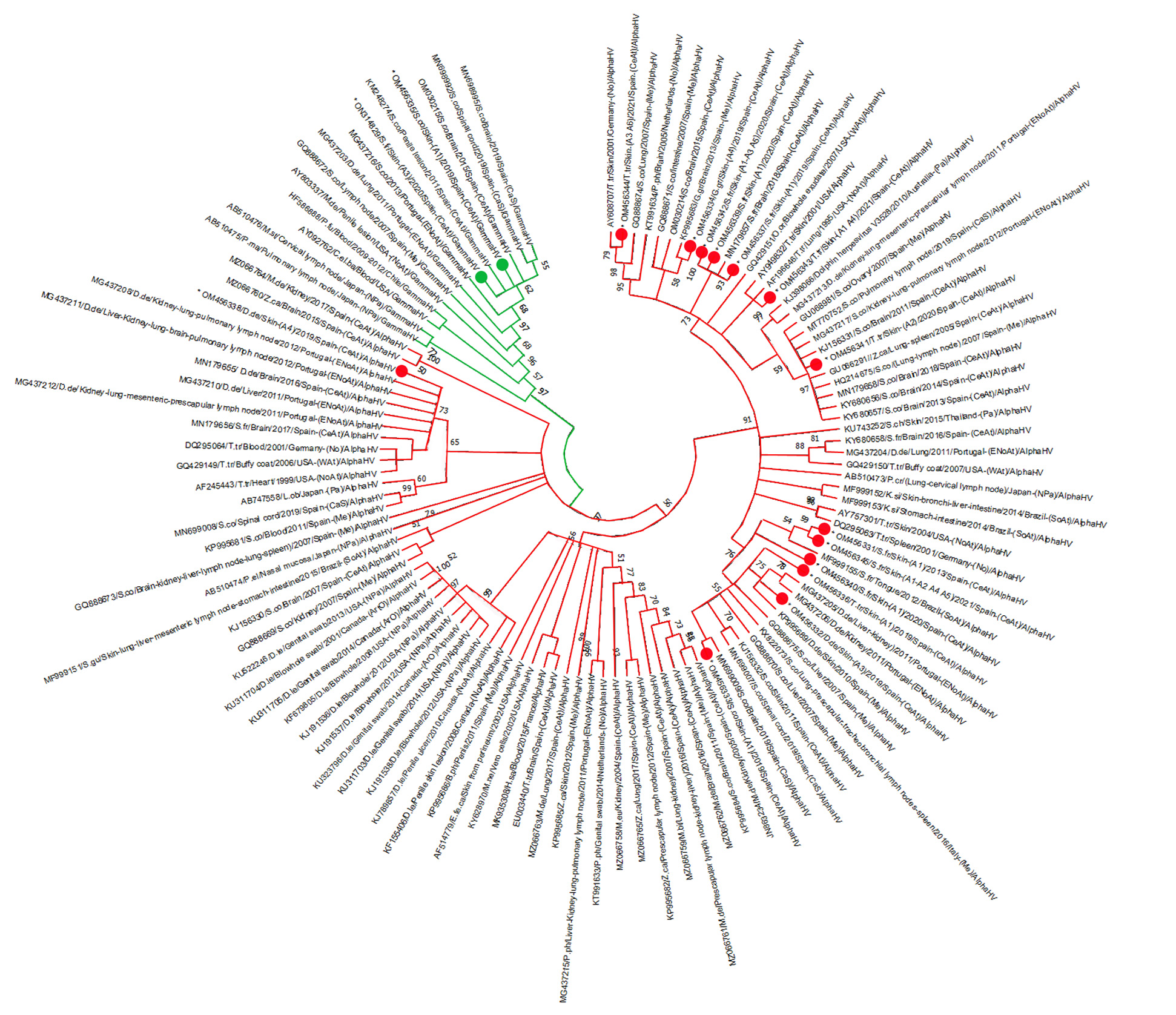

Supplement: Supplementary file 3 [file Image_2.JPEG]
